# B, N, and O Co‐Doped Nanoporous Activated Carbon With High Surface Area and Hierarchical Porous Structure for Enhanced Li‐Ion Battery and Supercapacitor Performance

**DOI:** 10.1002/smll.202513011

**Published:** 2026-04-17

**Authors:** P. A. Aleena, Rohan Bahadur, Vibin Perumalsamy, Solomon Ansah, R. K. Singh Raman, D. Sajan, Ajayan Vinu

**Affiliations:** ^1^ Global Innovative Centre for Advanced Nanomaterials (GICAN), College of Engineering Science and Environment (CESE) School of Engineering The University of Newcastle Newcastle New South Wales Australia; ^2^ Department of Physics Centre for Sustainable Energy and Environmental Technologies (CE^2^T) Bishop Moore College Alappuzha Kerala India; ^3^ Department of Mechanical & Aerospace Engineering Monash University Clayton Victoria Australia; ^4^ Department of Chemical & Biological Engineering Monash University Clayton Victoria Australia; ^5^ Department of Physics Centre for Sustainable Energy and Environmental Technologies (CE^2^T) Malabar Christian College Calicut Kerala India

**Keywords:** heteroatom, hierarchical porosity, Li‐ion battery, nanoporous carbon, supercapacitor

## Abstract

Nanoporous carbon materials with tunable physicochemical characteristics, such as high surface area, promising conductivity, and structural tunability, are attractive candidates for the design of high‐efficiency energy storage devices. In this work, B, N, and O co‐doped nanoporous carbon with high surface area and hierarchical pore structure has been synthesized through solid‐state activation of a mixture of boric acid, sucrose, and aminoguanidine using potassium citrate as the mild activating agent. The incorporation of B, N, and O not only introduces surface functionalities but also tailors the pore structure and surface area. The symmetric supercapacitor displayed an energy/power density of 34.32 Wh kg^−1^/599.99 W kg^−1^, respectively, with 100% cyclability up to 10,000 cycles. Further, when employed as anodes for lithium‐ion batteries (LIBs), the material exhibits an exceptional specific capacity of 1606.3/1415.2 mA h g^−1^ at 0.05/0.1 A g^−1^, which is an eight‐fold increase in the capacity compared to bare nanoporous carbon. Further, the ex situ SEM, TEM, EIS, and XRD measurements were carried out to analyze the material's structural changes post LIB cycling.

## Graphical abstract

The current research demonstrates co‐activation strategy for the development of heteroatom‐doped hierarchical nanoporous carbon for energy storage application, both in lithium‐ion batteries and supercapacitors.

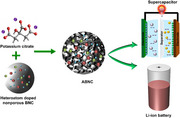

## 1 Introduction

The growing emphasis toward sustainable energy systems necessitates the advancement of cost‐effective, environmentally viable, and efficient energy storage solutions. Among energy storage technologies, supercapacitors and batteries are particularly promising due to their complementing properties, offering high power and energy density, respectively [1, 2, 3]. Nanoporous materials are viable electrode materials for energy storage due to their exceptional physicochemical properties, such as high specific surface area, tunable porosity, and superior electrical conductivity. Nanoporous materials with hierarchical porous structures are even more attractive for energy storage applications as they provide a unique combination of micro, meso, and macropores. These features are crucial to facilitate rapid ion transport, accommodating volume changes during charge–discharge cycles, which are key for enhancing the energy and power density [4, 5]. Porosity is commonly achieved through activation, wherein temperature, pressure, and the ratio of the activating agent. By varying these parameters or the type of activation, the nature of the porosity, including the hierarchical porous structure and the tunable specific surface area can be realized. While activating agents such as KOH and ZnCl_2_ are commonly utilized and are highly effective but corrosive, potassium citrate tribasic monohydrate (C_6_H_5_K_3_O_7_·H_2_O) (PC) offers a sustainable alternative, which serves a dual role both as an activating agent and a carbon source [6, 7].

While the carbon in these nanostructures offers electrical conductivity and PC assists in the creation of hierarchical porosity and improving the surface area and pore volume, the incorporation of heteroatoms is known to optimize the surface chemistry, electronic properties, and wettability, which are crucial for the efficient electron/ion transfer [8, 9, 10]. Previous reports have demonstrated that by virtue of the lone pair of electrons, N improves conductivity and imparts redox‐active sites. Boron, on the other hand, is electron‐deficient, and may assist in charge distribution and modification of the electronic band structure. Presence of oxygen in the material improves the surface hydrophilicity and enhances electrolyte ion accessibility. However, it may cause an increase in resistance or cause pore blockage due to excessive oxidation. Therefore, it is critical to control the amount of heteroatoms for optimized physicochemical and electrochemical properties. An integrated approach combining hierarchical porosity with the co‐doping of these heteroatoms can yield multifunctional carbons with high capacity, fast ion transport, and stable cycling. However, achieving this through an environmentally friendly route with simultaneous control over structure and composition remains challenging.

In this work, we report an integrated strategy that combines the introduction of a hierarchical porous structure with simultaneous co‐doping of multiple heteroatoms (B, N, and O) into nanoporous activated carbon to enhance its electrochemical performance in Li‐ion batteries and supercapacitors. To the best of our knowledge, the preparation of such materials with both hierarchical porosity and multiple heteroatom doping in a dual‐step synthesis has not been previously reported. Here, nanoporous activated carbon with a hierarchical porous structure co‐doped with multiple heteroatoms (B, N, and O) has been synthesized through the simple solid‐state activation of the mixture of B, N, C, and O sources using a mild activating agent, potassium citrate. The role of the reaction parameters and the amount of activating agent affecting the nature of the porous structure and the textural parameters of the final nanoporous activated carbon is exhibited. We also demonstrate how the synergy between hierarchical porosity and multi‐heteroatom doping significantly improves ion transport, structural stability, and electrochemical activity, leading to superior performance in both supercapacitors and Li‐ion batteries. Solid‐state carbon activation using potassium citrate is commonly reported. Current strategy utilizes the solid‐state activation approach along with the precise tuning of textural properties by varying the heteroatom precursors amount. Optimized material demonstrated a very high surface area compared to previous reports with a similar approach. Due to their high surface area, there are several reports demonstrating the potential of porous carbon as supercapacitor electrodes, however, the suitability for LIB anode fabrication is not well explored [6, 11, 12, 13]. The remarkably high capacity value makes the material an attractive candidate for LIB anode fabrication, which could overcome the volume expansion and poor stability of high‐capacity anode materials like Si and SnO_2_. The optimized material exhibits much better performance in both supercapacitor and Li‐ion battery compared to the carbon electrode without any doping and normal porous structure, offering a promising low‐cost platform for next‐generation high‐performance energy storage devices.

## 2 Materials and Methods

### 2.1 Material Synthesis

**FIGURE 1:**
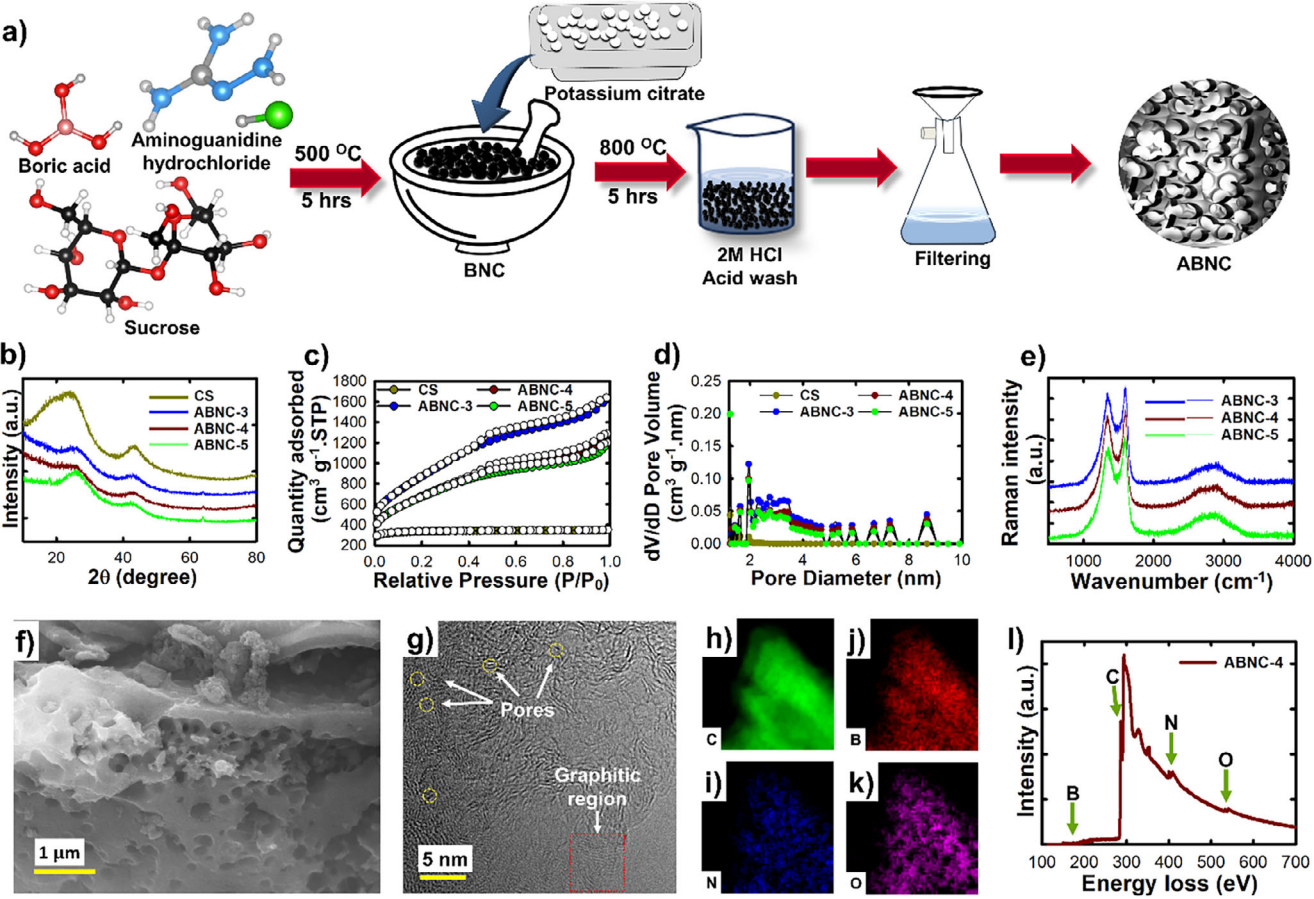
(a) Schematic illustration of the synthesis procedure for ABNC material, (b) XRD patterns, (c) BET N_2_ adsorption‐desorption isotherms, (d) Pore size distributions by NL‐DFT method, (e) Raman spectra of CS, ABNC‐3, ABNC‐4, ABNC‐5, (f) SEM image, (g) TEM image, EELS elemental mapping of (h) C, (i) N, (j) B, (k) O, and (l) EELS spectrum of ABNC‐4.

B, N, and O incorporated nanoporous carbon with different concentrations of N was prepared by facile two‐step pyrolysis followed by the solid‐state activation route. Boric acid (H_3_BO_3_) (BA), sucrose (C_12_H_22_O_11_), and aminoguanidine hydrochloride (CH_6_N_4_•HCl) (AG) were used as the precursors for B, C, and N, respectively. It may be noted that BA and sucrose also contain an ample amount of oxygen. 1 g of sucrose, 0.5 g of boric acid, and x g (x = 1, 2, 3, 4, and 5 g) of AG were mixed well in the agate mortar, and the mixture was pyrolyzed in the tubular furnace at 500°C for 5 h at a ramp rate of 5°C min^−1^ under continuous N_2_ flow. The composite is named as BNC‐x, where x denotes the amount of AG. In the second step for the solid‐state activation, the obtained BNC‐x materials were mixed thoroughly with PC and heated at a holding temperature of 800°C for 5 h at a ramp rate of 5°C min^−1^. After being taking out from the tubular furnace, materials were washed with 2 m hydrochloric acid (HCl), followed by DI water to remove the presence of potassium (K) and HCl subsequently. The obtained material was kept for drying at 100°C, and the activated materials are labelled as ABNC‐x, where x denotes the amount of N precursor. A control sample with only the carbon precursor and activation was also prepared utilizing the same procedure, and the material was represented as CS. Molar amounts of all the precursors used in the synthesis and the corresponding product yield is demonstrated in Table S1. Considering the relevance of the prepared materials in terms of performance, the materials ABNC‐3, ABNC‐4, ABNC‐5, and CS are considered in the main manuscript, and information about ABNC‐1 and ABNC‐2 is given in supplementary information as part of the optimization process. The synthesis procedure is illustrated in Figure 1a.

Conventionally, chemical activating agents such as KOH and ZnCl_2_ are commonly utilized, which are known to impart high surface areas, but are more corrosive in nature. PC is a less corrosive alternative that also acts as the carbon source [14]. During the activation process, at temperatures below 650°C, PC converts to potassium carbonate. At higher temperatures, potassium carbonate decomposes to potassium oxide (K_2_O) and carbon dioxide (CO_2_), which reacts with C to form CO. The volatiles evolved during activation may lead to the formation of interconnected pores [15]. The formation of CO during activation facilitates the creation of micropores. Further, the potassium causes swelling of the carbon‐based structures, leading to the formation of meso/macropores [16].

The reactions taking place during activation are as follows:

(1) $$2K_{3}C_{6}H_{5}O_{7\cdot }H_{2}O\to 3K_{2}{\mathrm{CO}}_{3}+9C+7H_{2}O$$

(2) $$K_{2}CO_{3}\to K_{2}O+CO_{2}$$

(3) $$CO_{2}+C\to 2\mathrm{CO}$$

(4) $$K_{2}O+C\to 2K+\mathrm{CO}$$

## 3 Results and Discussion

### 3.1 X‐Ray Diffraction (XRD)

The XRD patterns were measured to analyze the crystal nature of the materials. XRD patterns of activated ABNC materials exhibited a broad peak at ∼24°, which corresponds to the (002) lattice plane and another broad peak for (100) plane around 44° (Figure 1b), typical of graphitic structure. These peaks are much weaker than the peaks seen for the XRD pattern of CS. The BNC‐x compositions, on the other hand, showed narrower peaks at ∼24° (002) as compared to nonporous CS, but stronger than ABNC materials (Figure S1). As the amount of AG was increased, the shift to higher 2θ was observed, indicating a decrease in the interlayer spacing [17]. Post‐activation, the peak broadening indicated the formation of crystalline BCN/CN domains and transition toward a more disordered character due to the defect generation and structural deformation occurring in the presence of PC.

### 3.2 N_2_ Adsorption–Desorption Measurements

N_2_ adsorption‐desorption measurements were conducted to analyze the textural characteristics of the synthesized materials. All the ABNC materials exhibited a combination of type I and type IV isotherms according to the IUPAC classification, as illustrated in Figure 1c, and the detailed quantitative parameters are summarized in Table 1. In contrast, CS showed a type I isotherm depicting the microporous nature. Mesopores are identified by the H4‐type characteristic hysteresis loops appearing at higher relative pressures, indicating the presence of slit‐shaped pores [18]. These materials demonstrated elevated surface area and porosity, which may be attributed to PC as the activating agent. Among these compositions, a maximum surface area of 3324 m^2^ g^−1^ was observed for ABNC‐2. It is known that carbon plays a significant role in imparting the surface area to the material, therefore the surface area is reduced upon increasing the AG. This could be correlated with the formation of BCN and CN domains, leading to a decrease in the surface area. However, CS, prepared by activating the carbon precursor, exhibited a much lower surface area of 1319 m^2^ g^−1^ and a pore volume of 0.5 cm^3^ g^−1^. This signifies the role of the N and B in facilitating the activation process, leading to much higher surface areas and imparting mesoporosity in the material. N_2_ adsorption at low relative pressure corresponds to a type I isotherm, denoting the microporous nature of the nanoporous carbon in the absence of any heteroatoms. The incorporation of B, N, and O not only influences the chemical structure but also plays a key role in modulating textural characteristics of the material by facilitating the activation process and creation of mesopores. As can be seen, the incorporation of B, N, and O significantly increased the surface area from 1319 m^2^ g^−1^ to 3324 m^2^ g^−1^, and a noticeable improvement in pore volume of the materials was also observed upon doping.

**TABLE 1:** Textural parameters obtained from the BET N_2_ adsorption‐desorption measurements.

Material	SA_BET_ (m^2^g^−1^)	SA_micro_ (m^2^g^−1^)/%SA_micro_	PV (cm^3^g^−1^)	V_micro_ (cm^3^ g^−1^)/%V_micro_	PD (nm)	V_mes+mac_/V_t_ (%)
**ABNC‐1**	2745	2535/92	1.5	1.2/80	1.9	24.7
**ABNC‐2**	3324	2848/86	1.8	1.5/83	1.9	17.3
**ABNC‐3**	3024	1957/64	2.4	1.1/46	1.9	52.7
**ABNC‐4**	2707	1480/55	1.8	0.8/44	1.9	54.1
**ABNC‐5**	2434	1703/70	1.5	0.9/60	1.9, 1.2	36.7
**CS**	1319	1164/88	0.5	0.4/80	1.2	16.7

*Note*: **SA_BET—_** Specific surface area obtained using Brunauer–Emmett–Teller (BET) method, **SA_micro_** – micropore surface area derived from *t*‐plot, %**SA_micro_** – percentage contribution of micropore surface area to total surface area, **PV** – pore volume, V**_micro_** – micropore volume determined from *t*‐plot, %V**_micro_** – percentage contribution of micropore volume to total pore volume, **PD** – pore diameter, **V_mes+mac_ –** sum of pore volume of mesopores and macropores, and **V_t_ –** total pore volume.

The pore size distribution analysis using NL‐DFT and MP methods, illustrated in Figure 1d and Figure S2, respectively, revealed a combination of micro and mesopores in the ABNC materials, whereas CS showed a predominant micropore size distribution. The average pore size for CS was observed to be 1.2 nm, which could be correlated with its isotherm and dominant microporous character. The ABNC materials with higher nitrogen content depicted an average pore diameter of ∼1.9 nm. As observed from the isotherms, there was a significant improvement in the porosity of the material with the addition of B and N precursor, depicting the role of BA and AG. AG facilitates the process of activation and creation of larger pores, leading to a mix of micro and mesopores in the N incorporated materials. An increase in the pore size was observed with increased N loading amount. In fact, the hierarchical porosity emerges with the formation of BCN/CN by the generation of interconnected micro and mesopores.

Micropores assist electric double layer capacitance by offering an electrode‐electrolyte interface, whereas mesoporous channels enhance electrolyte diffusion, and macropores play their role by reducing ion diffusion length by serving as the electrolyte reservoir [19]. Hence, the combination of interconnected pores with hierarchical porosity makes the material suitable for more efficient electrochemical performance [20]. It is interesting to note that there is a significant effect on the textural parameters as the nitrogen precursor is tuned, with no change in the activating agent ratio. The appreciable surface area and hierarchical pore structure of the material provide a more efficient contact between electrode material and the electrolyte, which enhances the electrolyte ion diffusion, and assists in overall charge storage [21].

### 3.3 Raman Analysis

In the Raman spectra, the materials exhibited two prominent peaks around 1350 and 1590 cm^−1^, which represent the D and G bands, respectively (Figure 1e). These peaks are commonly observed in carbon‐based materials. Herein, the D band is associated with the disorders/defects in the sp^3^ hybridised carbon, whereas the G band is caused by the E_2g_ vibration in the sp^2^ hybridised carbon [22]. Another broad peak for the 2D band around 2800 cm^−1^ could be observed, substantiating structural disorder [23]. Emergence of this band may be attributed to the B and N incorporation along with the activation process, which causes an increase in the defect states within the material. The I_d_/I_g_ ratio was found to be maximum for ABNC‐4 (Table S2). A higher B and N content in the ABNC‐4 material could be correlated with the higher I_d_/I_g_ ratio, depicting the increase in defect states in this particular composition [24]. The I_d_/I_g_ decreases for ABNC‐5, which may be corroborated with the XRD data, reinstating the formation of BCN/CN domains leading to a more graphitic character.

### 3.4 Scanning and Transmission Electron Microscopic (SEM/TEM) Analysis

The morphology and porous characteristics of the material were confirmed using SEM and TEM imaging. SEM images of the ABNC samples exhibited a sponge‐like disordered structure resulting from the activation process (Figure 1f; Figures S3 and S4). In contrast, the CS structure has a flat surface with minimal surface deformity. This aligns well with the surface area and porosity analysis from the N_2_ sorption measurements. On increasing the N content, the flake‐like structure transitions to two‐phase structure, suggesting the presence of BCN/CN domains for compositions ABNC‐1 to ABNC‐4. However, an agglomerated structure was seen for ABNC‐5. The disordered hierarchical porous structure of the material was analyzed using TEM images. As observed from the TEM images (Figure 1g; Figure S5a,b), the prepared materials have localized graphitic microcrystalline domains (highlighted by red rectangle in Figure 1g), which provide the material with improved electric conductivity [25]. This is in agreement with the distribution of graphitic carbon as evident from XPS results. TEM images of CS (Figure S5c,d) demonstrate a turbostratic carbon structure with an amorphous nature. CS demonstrates abundant microporosity, which is confirmed through the BET measurements. Brighter spots indicate the abundance of micropores and mesopores within the material, which assist in the charge storage and ion transport [26, 27].

### 3.5 Energy Dispersive X‐Ray Spectroscopy (EDX)

Elemental composition of the ABNC‐4 and CS was examined using EDX spectroscopy. CS consists of C and O with atomic percentages of 89.76 and 10.24, respectively. In comparison, heteroatom‐doped ABNC‐4 contains C, N, and O with atomic % of 74.75, 11.69, and 13.56, respectively, whereas B was not detected owing to its low concentration. This analysis reveals the predominantly carbonaceous composition of the material.

### 3.6 Electron Energy Loss Spectroscopy (EELS)

EELS is an efficient method to quantify B in the material, as XPS is not sensitive enough for this element. The elemental mapping and its corresponding EELS spectrum for ABNC‐4 are shown in Figure 1h–l, confirming the contribution from B, N, C, and O in the optimized material [28]. The atomic % of B, N, C, and O in ABNC‐4 is calculated to be 3.8±0.2, 4.7±0.3, 84.2±0.8, and 7.3±0.5%, respectively. The high carbon content may be attributed to a combination of sucrose and PC. Comparing this with the XPS analysis (Table S2), a discrepancy was seen for the reasons mentioned above. However, it is noteworthy that the trend is similar. For example, for the composition ABNC‐4, the atomic % of B, N, C, and O was estimated from XPS to be 0.18, 3.85, 91.60, and 4.36, respectively. The N species bond easily with carbon at lower temperatures up to 600°C, however, higher temperatures are required for the B─N bonding, therefore, the N is observed to be higher than B. Even though covalently bonded heteroatoms remain unaffected by acid washing with 2 m HCl, loosely bound boron species like B─O can be removed due to its acid solubility [29]. This may cause the reduced B presence in the final material.

### 3.7 Fourier Transform Infra‐Red (FTIR) Spectroscopy Analysis

Chemical bonding and the presence of the functional groups in the prepared nanomaterials were investigated using FTIR spectroscopy (Figure S6). The band observed around 1581 and 1227 cm^−1^ can be attributed to C═C and C─O stretching bonds, which arise from the activation process [30]. Further, a broad band observed at 3414 cm^−1^ indicates the presence of the O─H bond [31].

### 3.8 Thermogravimetric Analysis (TGA)

Thermal behavior of the materials is evaluated using TGA (Figure S7). Initial weight loss exhibited by all the materials below 200°C is due to the desorption of adsorbed water [32]. Activated carbon without heteroatoms showed higher thermal stability, and with the incorporation of heteroatoms, weight loss increased, showing higher loss for ABNC‐5. Gradual weight loss over the temperature is from the slow decomposition of residual heteroatoms. Mass loss observed around 250°C is due to the decomposition of C─O bonds [33]. The presence of sp^2^ carbon networks offers the material higher thermal stability, and therefore, no sudden decomposition of the materials was observed within the temperature range of 35 to 800°C.

### 3.9 Contact Angle Measurements

To have further insight into the effectiveness of heteroatom doping for improving the wettability of the material, dynamic water contact angle measurements were performed. From the measurements using milli‐Q water, as illustrated in Figure S8, the materials exhibited a descending wettability trend as ABNC‐4 > ABNC‐3 > ABNC‐5 > CS. It is evident that ABNC‐4 is more hydrophilic and CS is hydrophobic. These results substantiate that the heteroatom doping increases surface affinity toward the liquid phase and leads to better electrolyte penetration. The intrinsic wettability promotes EDLC behavior with aqueous electrolyte and ensures better interaction with organic electrolytes as well [34].

### 3.10 X‐Ray Photoelectron Spectroscopy (XPS)

**FIGURE 2:**
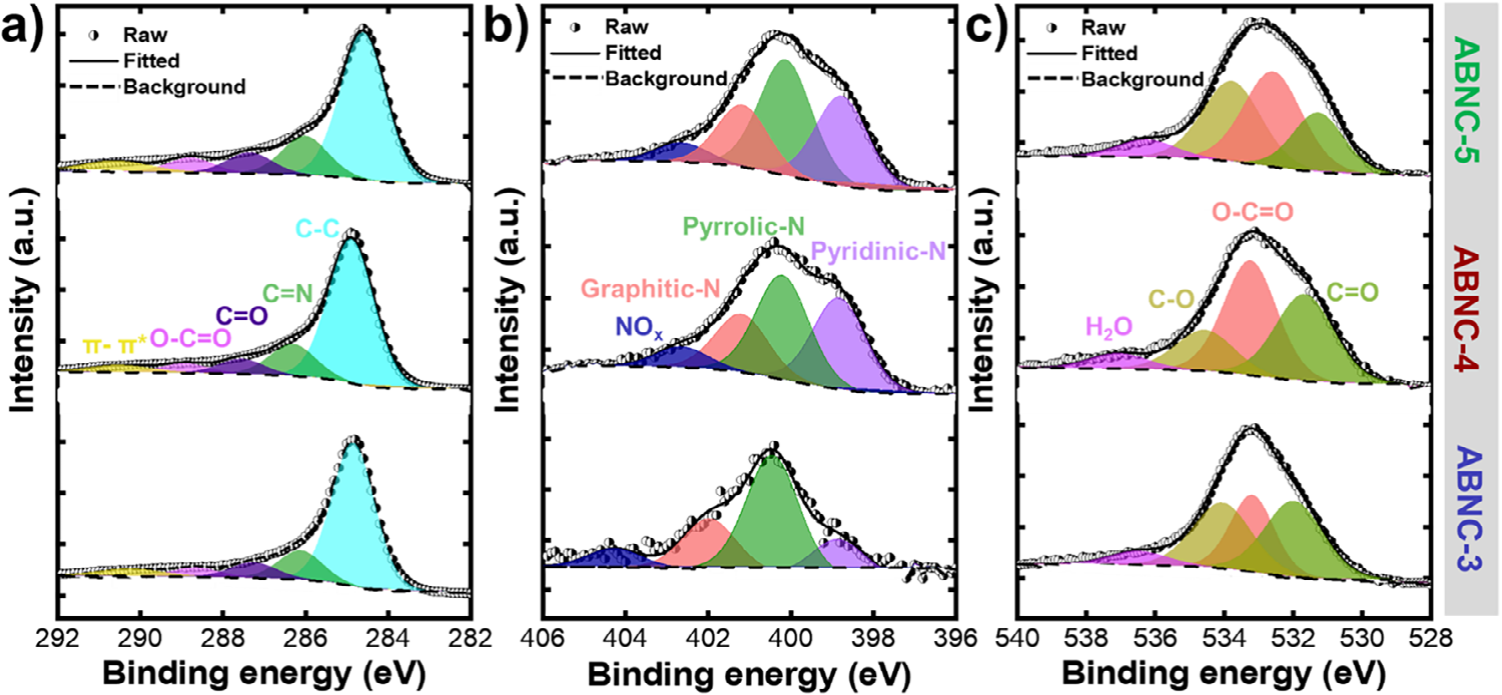
High resolution XPS spectra of (a) C1s, (b) N1s, and (c) O1s for the materials ABNC‐3, ABNC‐4, and ABNC‐5.

The quantitative estimation of the elements obtained from the survey spectra (Figure S9a) is given in Table S2. This aligns well with the trend observed in the EELS spectra, however, the discrepancy may arise as the XPS is primarily a surface‐sensitive technique, and it is not so sensitive for the estimation of B. The material consists predominantly of C, with a minor presence of B and N. Further, an ample presence of O may result from the precursors and activating agent. There was a clear increase in the nitrogen content as the AG was increased. The deconvoluted C1s spectra of ABNC‐4, shown in Figure 2a, exhibited five peaks at 284.9, 286.3, 287.6, 289.0, and 290.7 eV, which correspond to C─C, C═N, C═O, O─C═O, and π–π*, respectively [35, 36]. N1s spectrum exhibited three major peaks corresponding to pyridinic‐N/B─N, pyrrolic‐N, and graphitic‐N at 398.9, 400.3, and 401.3 eV, respectively (Figure 2b). Another peak at 402.7 eV could be observed, indicating the NO_x_ peak [37]. High‐resolution O1s spectra could be deconvoluted to four peaks at 531.7, 533.3, 534.6, and 537.2 eV (Figure 2c). The presence of these peaks indicates C═O, O─C═O, C─O, and adsorbed H_2_O [38]. The B1s spectra exhibited three peaks in the range of 190.8‐194.5 eV, which may imply the presence of B─C, B─N, and B─O bonds, albeit minimal, as the spectra were seen to be noisy and therefore were not further investigated (Figure S9b–d) [39]. The contribution arising from each bond is highlighted in Figure S10. In the C1s spectra, ABNC‐4 displayed the maximum amount of sp^2^ C contribution, followed by ABNC‐3, which is critical for the conductivity due to the π‐electron delocalization. Also, as the N was increased, the intensity of the C═N peak increased. Similarly, in ABNC‐4, the pyridinic/B─N contribution was observed to be the maximum in comparison, signifying the role of the B─N intercoupling in enhancing the charge transfer. The discrepancy between the XPS and EELS signifies the oxygen‐rich surface, which leads to thicker SEI formation and low first‐cycle CE. On the other hand, the reversible storage may be attributed to the hierarchical porosity.

### 3.11 Analysis of ABNC Materials as Supercapacitor Electrode

**FIGURE 3:**
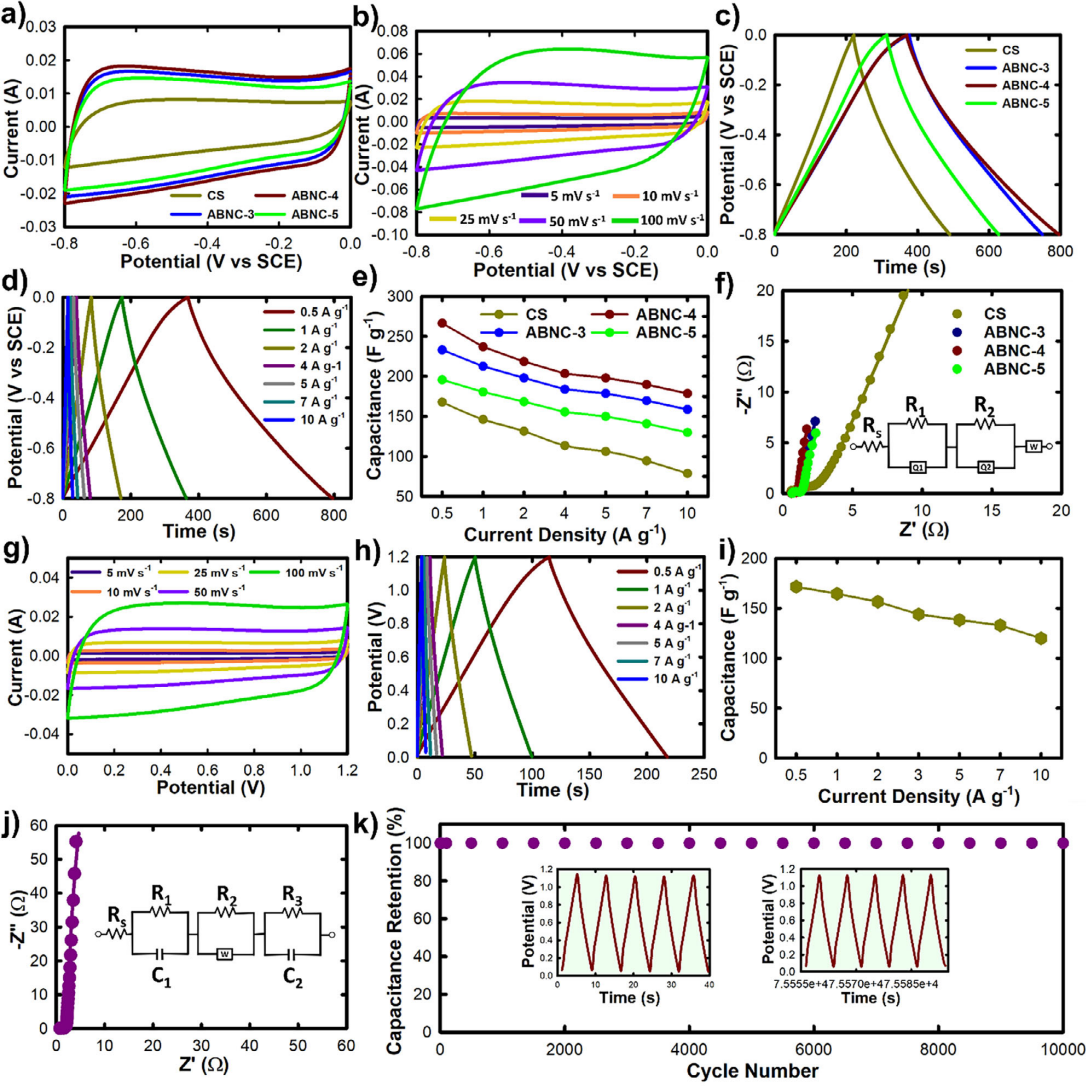
Supercapacitor measurements (a) CV curves for the materials at a scan rate of 25 mV s^−1^, (b) CV curves of ABNC‐4 at various scan rates, (c) GCD curves for all the materials at a current density of 0.5 A g^−1^, (d) GCD curves of ABNC‐4 at different current densities, (e) Comparison of specific capacitance with the current density, (f) Nyquist plot for CS, ABNC‐3, ABNC‐4 and ABNC‐5 and the corresponding circuit diagram used for fitting. Two electrode supercapacitor measurements of ABNC‐4 (g) CV curves at scan rates of 5–100 mV s^−1^, (h) GCD curves for the current densities 0.5–10 A g^−1^, (i) Variation of specific capacitance with current density, (j) Nyquist plot with the equivalent circuit, (k) Capacitance retention for 10 000 cycles at a current density of 5 A g^−1^ (inset: Initial and final five cycles of GCD at 5 A g^−1^ during the cycling).

Supercapacitor performance of the materials was analyzed using a three‐electrode setup with saturated calomel electrode (SCE) and Pt as the reference and counter electrode, respectively. For the working electrode, ABNC was mixed with acetylene black, which acts as the conducting carbon, and PVDF (solution in NMP) as the binder, and the mixture was loaded on the Ni foam. All the measurements were carried out with 3 m KOH alkaline electrolyte. The methods utilized for the electrochemical experiment are given in detail in Supporting Information. CV curves of all the materials exhibited quasi‐rectangular shape as shown in Figure 3a, which indicates the electric double layer capacitance (EDLC) nature of the charge storage. CV curves at various scan rates from 5–100 mV s^−1^ were recorded for ABNC‐4 (Figure 3b). The material retained a quasi‐rectangular shape even at high scan rates, which is an indication of the electrochemical stability of the material. A similar CV profile was also observed in ABNC‐3, whereas the CV curves deviated from the rectangular shape in the case of ABNC‐5 and CS denoting its poor EDLC capabilities due to its lower surface area (Figure S11a–c; Table 1). This was also supported by the GCD curves (Figure S11d–f).

As exhibited in Figure 3c, the triangular shape of the GCD confirmed the EDLC nature of the material. GCD curves at various current densities from 0.5 to 10 A g^−1^ for ABNC‐4 are shown in Figure 3d, and the comparative specific capacitance values are depicted in Figure 3e, Table 2, and Table S3. The material ABNC‐4 depicted the highest specific capacitance of 266.5 F g^−1^ at 0.5 A g^−1^, while maintaining a high capacity of 178.7 F g^−1^ at 10 A g^−1^. GCD curves at different current densities from 0.5 to 10 A g^−1^ for the other compositions are given in Figure S11d–f. As can be seen, the material CS only displayed a capacity of 167.9 F g^−1^ at 0.5 A g^−1^. The increase in specific capacitance was evident after B and N incorporation, in comparison to the CS material. On increasing the current density from 0.5 to 10 A g^−1^, ABNC‐4 exhibits rate capability of ∼67%, whereas CS has a lower value of around 47%.

**TABLE 2:** Specific capacitance values for the prepared materials and symmetric supercapacitor in the two‐electrode configuration at different current densities and the respective resistance values obtained from EIS fitting.

Current density (A g^−1^)	Capacitance (F g^−1^)				
	ABNC‐3	ABNC‐4	ABNC‐5	CS	2‐Electrode
0.5	233.1	266.5	195.8	167.9	171.6
1	212.7	236.9	180.7	146.2	164.7
2	198.2	218.7	168.5	131.5	156.7
4	184.0	203.5	155.5	113.5	144.0
5	178.8	198.1	150.0	106.3	138.3
7	169.8	189.9	140.9	94.5	133.0
10	158.7	178.7	130.0	78.7	120.0
Rate capability retention at 10 A g^−1^	68%	67%	66%	47%	70%
R_s_ (Ω)	0.64	0.60	0.64	0.81	—
R_ct_ (Ω)	0.57	0.37	0.57	1.14	—

The resistance of the material is an important parameter that accounts for the material's ability to facilitate ion diffusion and was analyzed using electrochemical impedance spectra (EIS) in the frequency range of 0.1 Hz to 10^5^ kHz. Nyquist plot for the materials along with their equivalent circuit is given in Figure 3f. Electrolyte resistance on the surface of porous electrode/series resistance is determined from the high‐frequency range, and the diffusion‐controlled double‐layer capacitance is evaluated from the low‐frequency region. The resistance values obtained using the circuit diagram fitting are given in Table 2. Semicircles in the middle frequency region represent the ion‐transport resistance between the interface of the electrode surface and the material. The smaller semicircle of ABNC‐4 confirmed the smaller charge transfer resistance (R_ct_), which could be confirmed using the values obtained from fitting (Figure S12). The charge transfer resistance (R_ct_) of ABNC‐4 was 0.37 Ω, significantly lower than CS (1.14 Ω), demonstrating increased charge transport. The significant difference in the semicircle radius in the high frequency region and the steeper line in the low frequency region compared to the CS demonstrates the effectiveness of B, N, and O coordination. The presence of lone pair of electrons from N increases the conductivity of the material, thereby leading to a decrease in resistance. Therefore, an enhancement in the supercapacitive performance due to the incorporation of B and N could be clearly observed. The electron deficient B can interact with OH^−^ ions present in alkaline electrolytes, supporting the higher performance [40]. On the other hand, the incorporation of O helps in improving wettability and hydrophilicity [41]. Along with the heteroatom incorporation, textural characteristics such as the high surface area and pore volume provided abundant electrochemically active sites. While the porosity of the materials generally facilitates the electrolyte penetration and leads to faster ion kinetics, mesopores in particular enhance the ion diffusion within the electrolyte, which effectively contributes to high rate capability [42]. It is important to note, however, that the optimal amount of O is crucial for better performance, as higher amounts can lead to adverse effects such as ion transport resistance and blockage of micro pores [43]. This was reflected in ABNC‐5 material, which registered a lower specific capacitance than that of ABNC‐4, caused by the change in the resistance due to higher O content in the former sample.

### 3.12 Symmetric 2‐Electrode Configuration

A symmetric two‐electrode supercapacitor using CR2032 coin cell setup with ABNC‐4 was fabricated, and the measurements were conducted in the potential window of 0–1.2 V. CV curves at various scan rates from 5–100 mV s^−1^ are given in Figure 3g. Quasi‐rectangular nature of the CV curves and the near triangular nature of the GCD curves indicate that the capacitance is governed by EDLC mechanism (Figure 3h). As shown in Figure 3i, the symmetric supercapacitor exhibited a high capacitance of 171.6 F g^−1^ at 0.5 A g^−1^ and retains a specific capacitance of 120 F g^−1^ at a higher current density of 10 A g^−1^. Rate capability of ∼70% at higher current density underlines the high electrochemical stability of the material. The resistance from the EIS demonstrates a small semicircle depicting the low resistance of the two‐electrode supercapacitor device (Figure 3j). The steeper line in the EIS curve represents the lower Warburg impedance, depicting efficient ion diffusion. The fabricated symmetric supercapacitor device exhibited an energy density of 34.32 Wh kg^−1^ and a power density of 599.99 W kg^−1^, comparable to reported works.

Cyclic stability of the material in the symmetric device was explored after 10 000 charge–discharge cycles at 5 A g^−1^, which demonstrated a capacitive retention of ∼100% (Figure 3k). As observed from the initial and final five charge–discharge curves, there is no apparent change (Figure 3k: inset). The possible reason for this high stability could be attributed to the presence of a 3D network of interconnected pores with a combination of micro‐and mesopores in the ABNC‐4, which could assist in rapid ion diffusion and enhancement in long‐term stability. A comparison of the specific capacitance values for similar materials is tabulated in Table S4, which clearly demonstrates the superior stability of the prepared material. The potential of heteroatom‐doped porous carbon materials with precisely tuned porosity and a certain degree of graphitization is obvious from these reports. To have an idea about the suitability of the material, the performance was compared (Table S4) with other promising materials like N‐doped graphene oxide decorated with copper ferrite and MXene/carbon nanotube composite, which are reported to exhibit high specific capacitance and significant capacitance retention. Ragone plot (Figure S13) provides a holistic view regarding the performance of the symmetric supercapacitor in comparison with other previously reported works on porous carbon‐based and other promising materials. As evident from the Ragone plot, optimized material possesses appreciable energy and power density. These results substantiate the effectiveness of synthesized materials as an electrode material for a supercapacitor.

### 3.13 Analysis and Mechanistic Insights on ABNC as an Anode Material for LIB

**FIGURE 4:**
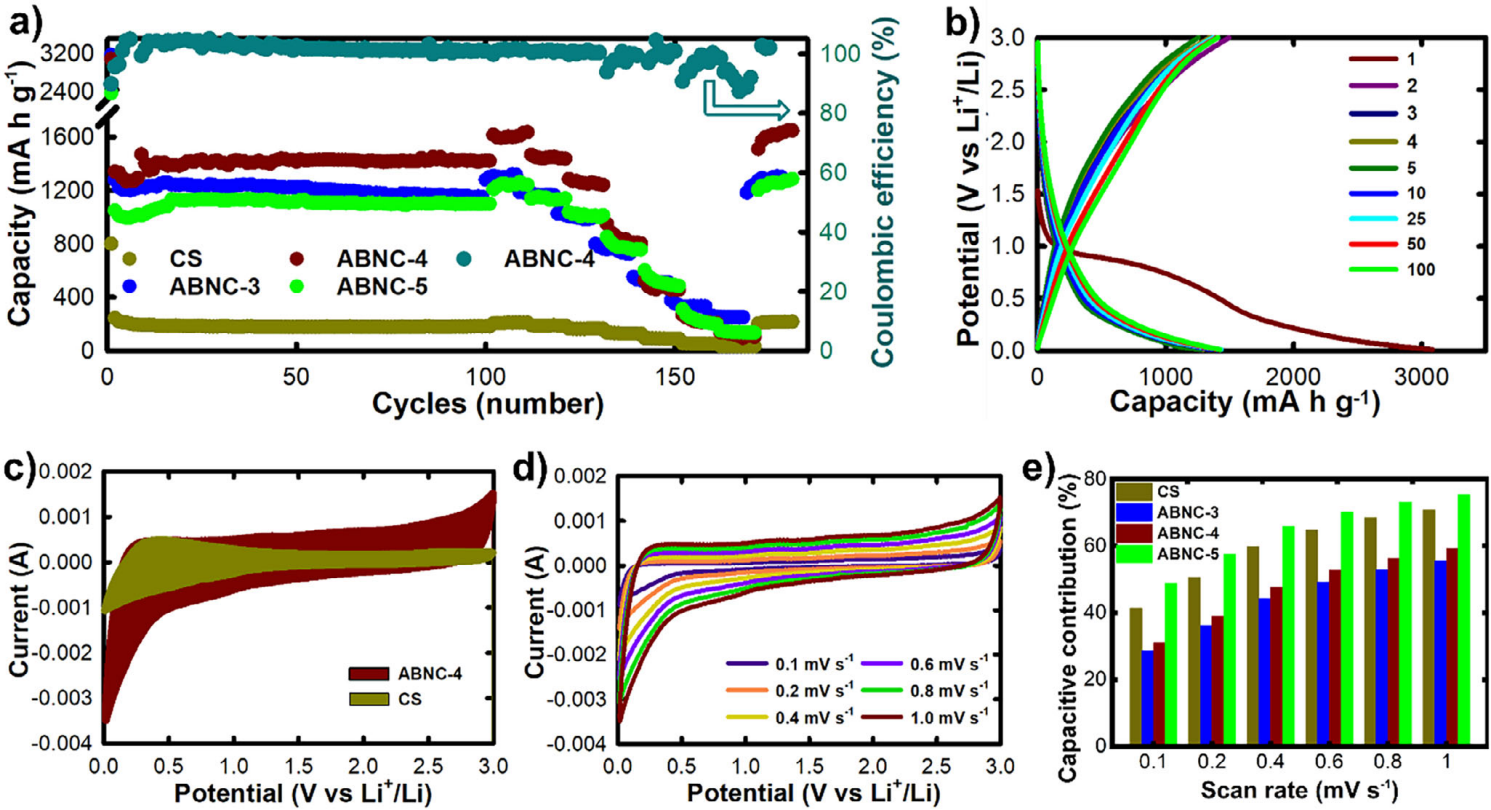
LIB measurements (a) Cycling performance of the materials at 0.1 A g^−1^ for 100 cycles followed by rate capability measurements at current densities of 0.05, 0.1, 0.2, 0.5, 1.0, 2.0, 3.0, 0.05 A g^−1^ for 10 cycles each, coulombic efficiency is shown for ABNC‐4 on the right y‐axis (dark cyan) (b) Galvanostatic charge–discharge profiles for ABNC‐4, (c) Comparative CV curves of ABNC‐4 and CS at 1 mV s^−1^, (d) CV curves of ABNC‐4 at different scan rates from 0.1 to 1.0 mV s^−1^, (e) Pseudocapacitive contribution for the materials at different scan rates.

The lithium storage capability of the material was also investigated using a CR2032 coin cell configuration wherein the ABNC material was used as the anode, and Li metal was utilized as the cathode. Detailed fabrication protocol regarding the coin cell fabrication is given in Supporting Information. Measurements were conducted in the voltage range 0.01 to 3.0 V. The specific capacity of the material could be analyzed using galvanostatic measurements at different current densities. 100 cycles were run at 0.1 A g^−1^, followed by 10 cycles each at 0.05, 0.1, 0.2, 0.5, 1, 2, 3, and then back to 0.05 A g^−1^. Significant cycling reversibility of the materials was demonstrated with the capacity going back to the initial capacity at 0.05 A g^−1^ and the rate capability analysis given in Figure 4a. At 0.05 A g^−1^, the materials ABNC‐1, ABNC‐2, ABNC‐3, ABNC‐4, and ABNC‐5 retain a capacity of 519.7, 948.7, 1284.8, 1606.3, and 1234.9 mA h g^−1^, respectively. For the optimized material, capacity measurements were obtained for three different coin cells to verify reproducibility, and the average values with corresponding error bars are shown in Figure S14. In comparison, CS demonstrated a noticeably low capacity of 213.2 mA h g^−1^ at 0.05 A g^−1^, highlighting the role of the heteroatoms in imparting certain redox functionalities and therefore obtaining higher energy density. ABNC‐4 exhibited energy and power density of 1990.5/158.2 Wh kg^−1^ and 62/3979.1 W kg^−1^ at current densities of 0.05 and 3 A g^−1^, respectively. Comparison of capacity for all materials is given in Table S5. As evident from the data, the structural modifications introduced by the B, N, and O coordination to C lead to almost an eight‐fold increment in capacity. Charge–discharge profiles are illustrated in Figure 4b and Figure S15.

Tunable porosity and high surface area are the crucial properties of porous carbon, which lead to superior performance compared to graphite when applied as the anode material for Li ion battery. There are reports on activated porous carbon‐based materials having a capacity >1200 mA h g^−1^ even without significant redox reactions. The favorable outcomes of high surface area and the presence of micro‐/meso‐pores in enduring better electrolyte penetration and ion transport are reported [44, 45]. Higher theoretical capacities of 2783–3032 mA h g^−1^ were proposed for microporous carbon, which demonstrate a 7–8 fold increase than graphite [46]. Incorporation of heteroatoms provides active sites for ion storage and significantly enhances the electrochemical performance by a capacitive‐dominating storage mechanism [47]. Multiple reports underline the fact that heteroatom doping is an effective strategy to increase the capacity of porous carbon beyond graphite by lowering the ion diffusion barrier and improving wettability [48]. Among them, nitrogen is considered, as a suitable choice for doping, and N‐doped carbon is reported as a substitute for graphite for LIBs [49]. Lithium intercalation in N‐doped carbon is guided through a combination of diffusion process and capacitive process that includes surface redox pseudocapacitance [50, 51].

Lithiation/delithiation processes were explored further using differential capacity (dQ/dV) profiles of first, second, and 100^th^ cycles (Figure S16). The strong reduction peak below 0.2 V in the initial discharge is attributed to electrolyte decomposition and the formation of the SEI layer, which leads to irreversible Li consumption. A broad peak between 0.4 and 0.7 V is a characteristic peak for heteroatom‐doped porous carbon due to the reduction of surface functional groups within the carbon matrix. A sharp peak around ∼0.9–1.0 V can be generated from additional electrolyte decomposition and structural rearrangement of SEI. This peak disappears in subsequent cycles and is irreversible. For the plot corresponding to the discharging of cycle 2, a large peak near ∼0.1 V represents Li intercalation into the carbon structure, which is governed by Li adsorption and pore filling. Broad peak around ∼1–2.5 V during charging represents the gradual Li extraction from the surface adsorption sites and micropores [52, 53]. Pseudocapacitive and diffusion‐limited Li^+^ storage within the disordered carbon matrix and interconnected pores are represented by the broad peaks. These observations are consistent with the CV curves and kinetic studies discussed later [54]. Structural robustness and interfacial stability of the material are evident from the similar curves for the second and 100th cycles. Noticeable reversibility and negligible degradation are in agreement with the charge–discharge profiles.

Modification of C‐based structures for realizing high Li^+^‐ion storage capacity and rate capability is a long‐term focus of research, and the incorporation of B, N, and O is a cost‐effective strategy that holds certain promise in the modification of the electrochemically active sites of the material. The N atom in the material can serve as the electron donor, decrease the bandgap, and enhance the electronic conductivity, whereas the incorporation of B likely contributes to the electronic band structure of the material that will help to reduce the potential barrier of Li^+^ diffusion [55, 56]. On the other hand, the presence of O in the carbon lattice enhances the surface wettability, Li ion adsorption, and further supports the ion diffusion.

Therefore, the incorporation of B, N, and O leads to a synergistic effect by offering electron and hole carriers and guarantees increased reversible stability, rate capability, and cycling stability [57]. Discharge specific capacity of ABNC‐4 for the initial two cycles is 3083.8 and 1341.7 mA h g^−1^. Initial coulombic efficiency of ABNC‐4 is 90%, which stabilizes at 100% from the fifth cycle. Previous studies suggest that high surface area materials have a tendency to form a thicker solid electrolyte interface (SEI), which subsequently may have a negative effect on the initial coulombic efficiency and the initial irreversible capacitive loss [58]. However, the interconnected pores in our materials help to improve the effective surface area, leading to a more uniform SEI layer and therefore improved electrolyte infiltration. Interconnected hierarchical porous network also supports charge transport, high diffusion, and storage of Li^+^ ions, facilitated by the meso‐channels in the material. Further, the hierarchical porous structure also provides a buffer for the volume expansion and contraction during the charge–discharge process without decomposition [59]. The electrochemical Li^+^ storage could be broadly categorized using two mechanisms, namely diffusion‐controlled process and a capacitive contribution. Diffusion‐controlled and capacitive behavior can be determined using the following formula:

$$I\left(V\right)=k_{1}v+k_{2}v^{0.5}$$

Wherein, I(V), k_1_v and k_2_v^0.5^ denote the measured current at a particular potential, and the contribution from capacitive and diffusion‐controlled processes, respectively.

The mechanism of Li^+^ insertion/extraction was analyzed using CV curves at various scan rates in the range of 0.1–1.0 mV s^−1^. Less significant redox peaks appeared on the CV curve only for the initial cycles. The area under the curve for ABNC‐4 increased significantly compared to that of CS, which supports the findings from the capacity measurements (Figure 4c,d). Pseudocapacitive contribution of the materials was evaluated using CV curves at different scan rates. As evident from Figure 4e, the pseudocapacitive nature increased with scan rate due to the higher ion diffusion.

**FIGURE 5:**
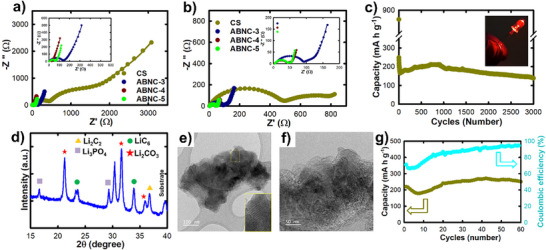
LIB measurements (a) Nyquist plots of the materials before cycling (inset: EIS data for only ABNC‐3, ABNC‐4, and ABNC‐5), (b) Nyquist plots of the materials after cycling **(inset**: EIS data of ABNC‐3, ABNC‐4, and ABNC‐5), (c) Cycling stability of ABNC‐4 coin cell for 3000 cycles at 3 A g^−1^ (inset: a red LED powered by the ABNC‐4 coin cell), (d) Ex situ XRD pattern of ABNC‐4 coin cell after stability test, (e,f) Ex situ TEM images of ABNC‐4 coin cell after stability test, (g) Cycling performance of the full cell at 0.1 A g^−1^ for 60 cycles, coulombic efficiency is shown on the right y‐axis (cyan).

Nyquist plots for all the materials before and after cycling are shown in Figure 5a,b, which give an insight into the resistive nature of the material. R_CTs_ of the materials were represented by the diameter of the semicircle in the high frequency region, and were observed to be less for ABNC‐4. The EIS data of ABNC‐4 after rate capability measurements are shown in Figure S17. The two semicircles observed after cycling may arise from the resistance offered by the SEI layer formation (R_SEI_) and the charge transfer resistance (R_CT_) between electrode and electrolyte, respectively. The circuit used for fitting the data and the corresponding resistance values are given in Figure S18 and Table S6. The R_CT_ is directly correlated with the resistance offered at the electrode‐electrolyte interface. It is worth noting that there was a significant reduction in R_CT_ values after B, N, and O incorporation. The R_CT_ drops from 503.69 to 23.7 for CS and ABNC‐4, respectively. Further, CS also demonstrates a significant SEI resistance, which might lead to decreased rate capability and capacitance retention. Charge transfer resistance of the material decreased after the charge–discharge cycles due to increased electrical conductivity and the Li^+^ insertion, enhancing the charge transfer capabilities. As the material was cycled, the pristine carbon structure converted into a suitable Li^+^ host, with interlayers and electrochemically active surface sites. Compared to heteroatom incorporated materials, CS possessed a higher charge transfer resistance underlining the effectiveness of the incorporation of heteroatoms into the carbon matrix for enhancing Li^+^ diffusion and storage.

The material ABNC‐4 showed a cycling stability of ∼56% at 3 A g^−1^ after 3000 cycles (Figure 5c). The interconnected porous structure is useful to mitigate the volume expansion during charge–discharge cycles, benefiting the long‐term stability. Ex situ XRD and TEM analysis were carried out to get an insight into the structural changes occurring during the charge–discharge. Ex situ XRD was carried out after cycling, and the results are shown in Figure 5d. These results indicate the formation of the compounds Li_2_CO_3_, Li_2_C_2_, LiC_6_, and Li_3_PO_4_, which arise due to the reaction of the Li^+^ with the carbon during the charge–discharge. Ex situ TEM images illustrated the morphology changes after battery cycling. Lattice observed in the TEM images (Figure 5e,f) with a d‐spacing of 0.42 nm corresponded to (110) plane of Li_2_CO_3_, which may also be identified as the predominant phase in XRD.

Structural and morphological changes occurring over the cycles were further analyzed from ex situ XRD spectra and SEM images recorded at 1st, 5th, 10th, and 100th cycles (Figure S19). Li compound formation, as identified from the XRD spectra, is identical to the one after stability measurements (Figure 5d). Morphological analysis reveals appreciable structural integrity of the material, even though surface roughening occurs (Figure S19b–e). Ex situ TEM images (Figure S20) demonstrate the evolution of the ABNC‐4 structure over the 100 cycles at 0.1 A g^−1^. The structure after the first cycle shows ample reversibility, and some lithiated deposits could be observed on the ABNC‐4 structure (Figure S20a). Further, as the material is cycled further, significant changes are observed with the formation of thicker SEI layers. The SEI layer observed in cycles 5 and 10 are relatively of similar thickness denoting the stabilization of the structure after the first cycle (Figure S20b,c). After the 100^th^ cycle (Figure S20d), the material images demonstrate stacked graphitic layers within the carbon framework, confirming the stability and preservation of the structure post‐repeated cycling. As evidenced from the dQ/dV plots, decomposition of the electrolyte in the initial cycle results in irreversible capacity loss. The growth of SEI layer is clearly visible from the TEM image after the 100^th^ cycle. The interconnected porosity may be useful to accommodate the volume change and avoid structural collapse due to the lithium‐induced strain.

CV curve at 1 mV s^−1^ and EIS data of the coin cell after stability studies are given in Figure S21. The CV curve retains its shape after 3000 cycles denoting the material's stability. Cycling of ABNC‐4 coin cell in the voltage window 0.1–3.0 V was conducted (Figure S22). The cell exhibited a capacity of ∼175 mA h g^−1^ at 1A g^−1^, which is lower than the value observed within the wide potential window of 0.01–3 V. It is reported that for carbon‐based electrodes, the capacity below 0.5 V emerges from Li^+^ storage by intercalation, adsorption, or pore filling approach, whereas the capacity above this voltage represents capacitive charge storage on surface and edge sites [60]. Hence, the selection of an appropriate potential window is important. In porous carbon materials with hierarchical porosity, micropore filling contributes well to charge storage and takes place at near‐zero voltage [44]. As ABNC‐4 contains a micropore volume of 0.8 cm^3^ g^−1^, therefore, there is ample micropore in the structure leading to significant charge storage at lower voltage approaching zero. Charge storage by Li clustering occurring at low voltage is also being limited, and hence, a reduced capacity was observed [61]. Therefore, to access the complete micropore storage capacity, the voltage window up to 0.01 V was utilized.

To obtain a better understanding of the mechanism, ex situ measurements were conducted at different states of the charge–discharge process. Operando electrochemical impedance spectroscopy test was performed at potentials of 0.01, 1.5, and 3 V during charging, followed by 1.0 and 0.01 V during discharging, as shown in Figure S23a. On analyzing the semicircle radius, R_CT_ was observed to be minimum when the cell is fully charged. R_CT_ value represents the charge transfer resistance, and in the completely charged state, higher voltage and the active state of the electrochemical reaction led to a lower R_CT_ value. After complete discharge, the anode material goes back to the initial state, which confirms the reversible nature of charge–discharge [62]. The ex situ XRD (Figure S23b) depicts the formation of Li‐based compounds during charging and discharging. Even though the position of the peaks remains unchanged, a sharp decrease in the peak intensity is observed after complete discharge. These changes refer to the Li^+^ ion intercalation into the pores and carbon matrix [63]. Structural changes occurring during the charge–discharge process are reversible. Lithium deposition could be observed at different charge–discharge stages and the reversible nature of the material is observed from the SEM images (Figure S23c–f). Since porous carbon possesses a highly reactive surface in comparison to graphite, together with higher surface area, it can lead to significant electrolyte reduction and the formation of a thicker and heterogeneous SEI layer. One successful strategy to regulate the formation of SEI layer formation in porous carbon is the incorporation of surface functional groups or heteroatoms. It is well known that oxygen‐containing functional groups play a critical role in capacity. Oxygen is more prominently attached to the edge carbons of the lattice and within the voltage range 3.5 V to 1.5 V vs Li, C═O bond gets reduced to C─O single bond by Li^+^. Functional groups C─O─C will also react with Li ion, and these reactions may cause an increase the capacity [64, 65]. These faradaic reactions of oxygen result in increased capacity [66]. Polar anchoring sites generated by O atom improve electrolyte affinity and assist in Li ion adsorption and support uniform SEI formation [67]. Electron‐deficient B atoms make nearby carbon atoms electrophilic. While playing a role in Li^+^ adsorption, it controls electrolyte reduction and encourages inorganic‐rich SEI formation [68, 69]. B doping is considered an effective strategy to control electrolyte decomposition and the formation of a uniform SEI layer. The robust nature of this layer prevents further decomposition [70]. Pyridinic N ensures strong Li bonding, graphitic N enhances electronic conductivity, and pyrrolic N regulates defect‐mediated reactions. An electron‐rich N atom generates active defect sites and facilitates faster and more uniform SEI nucleation [71]. In brief, heteroatom incorporation may be a useful method for the formation of thin, uniform, ion‐conductive SEI and leads to better pore accessibility and long‐term stability.

A full cell was fabricated and analyzed for the application performance of the material. As depicted in Figure 5g, the cell exhibited a specific capacity of around 250 mA h g^−1^ at 0.1 A g^−1^. Inspired by the appreciable performance of the material, a LIB coin cell fabricated with ABNC‐4 was used to operate a rotating fan. The cell could rotate it for 15s as illustrated in the Video S1, and a red LED could be lit by the cell (Video S2; Figure 5c inset). The capacity of the material is compared with similar materials and with high‐capacity anode materials to get an insight into the potential of the prepared material (Table S7). ABNC‐4 demonstrated capacity comparable to anode materials like Si and SnO_2_, which have a significantly higher theoretical capacity. While offering higher capacity, the performance of these materials is often limited by volume expansion and poor cycling stability, as can be seen from the table. Interconnected porosity of the material accommodates the volume changes during the cycling and guarantees outstanding structural stability. Precisely tuned porosity in heteroatom‐doped porous carbon regulates the charge‐storage mechanism and offers remarkably high capacity in comparison to the previously reported state‐of‐the‐art materials [46].

These results substantiate the fact that the design of materials is crucial in obtaining high efficiency and electrochemically stable anode materials. We found that the interconnected porosity in the nanoporous activated carbon helps in accommodating volume expansion and contraction during charge–discharge and lithiation‐delithiation, leading to improved rate capability and stability. However, larger surface areas may sometimes be detrimental to the initial coulombic efficiency and cause electrolyte degradation, leading to lower cyclability and irreversible capacity loss. The presence of the combination of heteroatoms plays a crucial role in mitigating the side reactions, improving the wettability, enhancing the ion adsorption and diffusion, and further stabilizing the SEI, leading to superior rate performance. Therefore, it is imperative to find the right balance between surface area, nature of porosity and suitable surface functionalities. This work paves a path for developing dual‐function materials for supercapacitors and LIBs with high surface area and heteroatom incorporation.

## 4 Conclusions

The B, N, and O incorporated nanoporous activated carbon with a hierarchical porous structure and high specific surface area was synthesized through a simple solid‐state activation of the B, C, and N precursors using PC as a mild activating agent and carbon source. The use of nitrogen precursors shows a dual role of surface functionality in the nanoporous carbon along with porosity manipulation, which is optimized for superior electrochemical performance, both in supercapacitors and LIBs. The reaction parameters and the amount of the activating agent and the precursors were varied to fine‐tune the porous structure and the textural properties of the nanoporous activated carbons. EELS demonstrated a substantial amount of B, N, and O incorporation of 3.8±0.2, 4.7±0.3, and 7.3±0.5%, respectively, in the prepared nanoporous activated carbon, demonstrating the suitability of this process. In supercapacitors, a specific capacitance of 266.5 F g^−1^ was achieved at 0.5 A g^−1^ in a three‐electrode setup. In the symmetric supercapacitor coin‐cell, the material demonstrated ∼100% capacitance retention after 10 000 cycles, with an energy density of 34.3 Wh kg^−1^ at a power density of 599.9 W kg^−1^. Further, the LIB performance was investigated with a promising specific capacity of 1415.2/1606.3 mA h g^−1^ at 0.1/0.05 A g^−1^, and the full cell delivered a capacity of 250 mA h g^−1^ at 0.1 A g^−1^. The ex situ battery measurements revealed insights into the structural integrity of the material after battery cycling.

## Author Contributions

R.B. and A.V. conceived the idea. P.A. carried out the material synthesis, and energy storage performance evaluation with the help of R.B., D.S., and A.V. S.A., and R.K.S.R. carried out the XPS and Raman measurements. V.P. did the TEM and EELS measurement. P.A. wrote the manuscript. R.B. and A.V. finalized the manuscript.

## Conflicts of Interest

The authors declare no conflicts of interest.

## Supporting information




**Supporting File 1**: smll73416‐sup‐0001‐SuppMat.docx.

**Supporting File 2**: smll73416‐sup‐0002‐VideoS1.mp4.

**Supporting File 3**: smll73416‐sup‐0003‐VideoS2.mp4.

## Data Availability

The data that support the findings of this study are available from the corresponding author upon reasonable request.
