# Supplementary material for: B, N, and O Co‐Doped Nanoporous Activated Carbon With High Surface Area and Hierarchical Porous Structure for Enhanced Li‐Ion Battery and Supercapacitor Performance
Source: Small. 2026 Apr 17;22(32):e13011. doi: 10.1002/smll.202513011 (PMC13244419; doi:10.1002/smll.202513011)
Supplement: Supplementary file 1 — Supporting File 1: smll73416‐sup‐0001‐SuppMat.docx. [file SMLL-22-e13011-s003.docx]

**Supporting information**

**B, N and O Co-Doped Nanoporous Activated Carbon with High Surface Area and Hierarchical Porous Structure for Enhanced Li-Ion Battery and Supercapacitor Performance**

P. A. Aleena^a,b^, Rohan Bahadur^a^*, Vibin Perumalsamy^a^, Solomon Ansah ^c^, R.K. Singh Raman ^c,d^, D. Sajan^b,e^ and Ajayan Vinu^a^*

^a^Global Innovative Centre for Advanced Nanomaterials (GICAN), College of Engineering, Science and Environment (CESE), School of Engineering, The University of Newcastle, Callaghan, NSW 2308 Australia

^b^Department of Physics, Centre for Sustainable Energy and Environmental Technologies (CE^2^T), Bishop Moore College, Mavelikara, Alappuzha, Kerala 690110 India

^c^Department of Mechanical & Aerospace Engineering, Monash University, Clayton, Victoria, 3800 Australia

^d^Department of Chemical & Biological Engineering, Monash University, Clayton, Victoria, 3800 Australia

^e^Department of Physics, Centre for Sustainable Energy and Environmental Technologies (CE^2^T), Malabar Christian College, Calicut, Kerala 673001 India

**Corresponding author/s email**: [ajayan.vinu@newcastle.edu.au](mailto:ajayan.vinu@newcastle.edu.au)

[rohan.bahadur@newcastle.edu.au](mailto:rohan.bahadur@newcastle.edu.au)

**List of tables**

**Table S1:** Amounts of precursors used for synthesis and the corresponding product yields.

**Table S2:** Quantitative estimation of B, C, N, and O using XPS survey spectra and I_d_/I_g_ ratio determined from Raman spectra.

**Table S3:** Specific capacitance values of the ABNC-1 and ABNC-2 at different current densities.

**Table S4:** Literature comparison of specific capacitance of similar materials and their textural properties.

**Table S5:** Comparison of the stabilized specific capacity of materials at various current densities in LIBs.

**Table S6:** Comparison of resistance values obtained from fitting of Nyquist plots before and after cycling.

**Table S7:** Literature comparison of the capacity for some similar reported anodes in lithium-ion batteries.

**List of figures**

**Figure S1.** XRD patterns of non-porous materials.

**Figure S2.** Pore size distribution by MP method.

**Figure S3.** SEM images of **a)** CS, **b)** ABNC-3, and **c)** ABNC-5.

**Figure S4.** SEM images of **a)** ABNC-1 and **b)** ABNC-2.

**Figure S5.** TEM images of **a-b)** ABNC-4 and **c-d)** CS at various magnifications.

**Figure S6.** FTIR spectra of CS, ABNC-3, ABNC-4, and ABNC-5.

**Figure S7.** TGA analysis of CS, ABNC-3, ABNC-4, and ABNC-5.

**Figure S8. a)** Materials attached on carbon tape for contact angle measurements, **b)** Water contact angle images of materials with Milli-Q water, and **c)** measured angles with standard deviations (*p < 0.05, p <0.01, ns – not significant).

**Figure S9. a)** XPS survey spectra for ABNC-3, ABNC-4, and ABNC-5, High resolution B1s XPS profiles of **b)** ABNC-3, **c)** ABNC-4, **d)** ABNC-5.

**Figure S10.** Contribution of bonds from **a)** N1s, **b)** C1s and **c)** O1s spectra for ABNC-3, ABNC-4, ABNC-5.

**Figure S11.** CV curves at scan rate 5–100 mV s^-1^ for **a)** CS **b)** ABNC-3 **c)** ABNC-5, and GCD curves for the current densities 0.5–10 A g^-1^ for **d)** CS **e)** ABNC-3 **f)** ABNC-5.

**Figure S12.** Nyquist plot of ABNC-4 at 0.1 - 10^5^ Hz frequency (**inset:** EIS at 0.01Hz – 10^5^ Hz).

**Figure S13.** Ragone plot comparing the symmetric supercapacitor device with others in literature.

**Figure S14.** Average capacity of ABNC-4 obtained from three different coin cells with corresponding error bars.

**Figure S15.** Galvanostatic charge-discharge profiles for **a)** CS, **b)** ABNC-3 and **c)** ABNC-5.

**Figure S16. a)** Galvanostatic charge-discharge profiles and **b)** corresponding differential capacity (dQ/dV) curves of cycle 1, 2, and 100.

**Figure S17.** Nyquist plot of ABNC-4 LIB coin cell after rate capability measurements.

**Figure S18.** Equivalent circuit diagram for Nyquist plots for half-cell **a)** before and **b)** after cycling.

**Figure S19a. a)** Ex-situ XRD spectra of ABNC-4 anode after 1^st^, 5^th^, 10^th^, 100^th^ cycles, Ex-situ SEM images after **b)** 1^st^, **c)** 5^th^, **d)** 10^th^, **e)** and 100^th^ cycles.

**Figure S20a.** Ex-situ TEM images of ABNC-4 anode after **a)** 1^st^, **b)** 5^th^, **c)** 10^th^, and **d)** 100^th^ cycles.

**Figure S21. a)** CV curve at 1 mV s^-1^ and **b)** Nyquist plot of ABNC-4 LIB coin cell after stability measurements.

**Figure S22a.** Cycling of ABNC-4 coin cell at 1 A g^-1^ in the potential window 0.1 to 3V.

**Figure S23. a)** Nyquist plots recorded at different stages of charge-discharge, **b)** XRD spectra at **(i)** 1.5 V, **(ii)** 3 V during charging and **(iii)** 1.0 V, **(iv)** 0.01 V during discharging, SEM images **c)** at 1.5 V during charging, **d)** after complete charging, **e)** at 1.0 V during discharge, and **f)** after complete discharge.

***Materials***

Boric acid (BA) (99.999%), aminoguanidine hydrochloride (AG) (≥ 98.0%), and potassium citrate tribasic monohydrate (PC) (≥ 99.0%) were purchased from Sigma-Aldrich. Sucrose (> 99.5%) and hydrochloric acid (HCl) (36%) were acquired from chem-supply and TCI respectively.

***Characterization Techniques***

Structural properties of the materials were analyzed using powder X-ray diffraction (XRD) conducted by PANalytical Empyrean, by irradiating the samples with Cu Kα radiation having λ=1.5405 Å generated at operating current and voltage of 40 A and 40 V, respectively. N_2_ adsorption-desorption measurements for the analysis of surface area and porosity were conducted using Micromeritics ASAP 2420 Analyzer under liquid nitrogen temperature of -196 °C. Degassing of the samples at 200 ºC for 12 hours was conducted before the analysis. Surface morphology of the materials was studied using Scanning electron microscopy (SEM) images from JEOL JSM-7900F at an operating voltage of 10 kV. Elemental composition of the materials was examined by energy dispersive X-ray spectroscopy (EDX) attached to the same instrument. Transmission electron microscopy (TEM) measurements are carried out using JEOL JEM-F200 multi-purpose electron microscope. Electron energy loss spectroscopy (EELS) measurements are carried out using Jeol JEM-ARM300F2 GRANDARM Atomic Resolution Analytical Microscope.

Raman spectroscopic measurements were recorded with the help of WITec Raman alpha300 Spectrometer with 600 grooves mm^−1^ grating and a 532 nm laser with 2 mW power. Each Spectrum is an average of 10 accumulations, where each accumulation is recorded for 5 s of integration time. Chemical composition of the materials is evaluated by XPS spectroscopy measurements using the instrument ESCA+ Omicron Nanotechnology. For XPS analysis, quantitative estimation of elements was obtained from integrating peak areas using CasaXPS software after Shirley background subtraction. Charge correction was performed by fixing C1s peak at 284.8 eV. High resolution spectra were deconvoluted using Gaussian-Lorentzian functions. During XPS peak fitting process, particular care was taken to ensure that the FWHM values of individual peaks closely match the reported standard values.

Perkin Elmer Frontier spectrometer was used for the Fourier transform-infrared spectroscopy (FT-IR) measurements for the in-depth analysis of functional groups and molecular bonds by potassium bromide (KBr) pellet method in which material together with KBr were grinded well for preparing the pellets. The thermogravimetric analysis (TGA) of the materials was analyzed using PerkinElmer model STA-8000 analyzer in nitrogen atmosphere within the temperature range of 35 to 800°C. 5-10 mg of the material was loaded into an alumina crucible for the measurements. Contact angle measurements were performed using Kruss drop analyzer DSA25KBUS. Milli-Q water collected from Sartorius arium mini plus is used for the analysis. All powder-based samples (CS, ABNC-3, ABCN-4, and ABCN-5) were attached to black carbon tape and subsequently adhered to glass slides for water contact angle characterization. Measurement for each sample is repeated three times with 20 μL water drops and the value of contact angle with error values is determined.

***Supercapacitor measurements***

Supercapacitor performance of the samples was analyzed using CHI 760E electrochemical workstation. ABNC-x was mixed well with conductive acetylene black (AB) and binder polyvinylidene fluoride (PVDF) dissolved in n-methylpyrrolidine (NMP) in the ratio of 65: 25:10 in a mortar pestle. Obtained mixture was coated on Ni foam, which serves as the current collector, and dried at 60°C for 12 hours. In three-electrode configuration, measurements were conducted in 3M KOH electrolyte solution with SCE and Pt rod as reference and counter electrodes respectively. Cyclic voltammetry (CV) and galvanostatic charge-discharge (GCD) measurements were carried out in the potential range of −0.8 to 0 V. Electrochemical impedance spectroscopy (EIS) was recorded for the frequency range of 10^−2^ – 10^5^ Hz.

Two electrode measurements were conducted using a CR2032 coin cell setup where material coated on Ni foam as previously described, served as the working electrode and counter electrode in the symmetric supercapacitor. These were separated using whatman filter paper soaked in 3M KOH electrolyte. A wider potential window of 0 to 1.2 V was applied for CV and GCD measurements.

Specific capacitance of the material in the three-electrode setup was calculated from the GCD data using the equation

C (F g^-1^) = $\frac{I\Delta t}{m\Delta V}$ (1)

Where, I (A) denote discharge current, $\Delta t$ (s) is the discharge time, m (g) is the mass of the active material, and $\Delta V$ is the potential window.

Capacitance (C), energy density (E) and power density (P) of the material for the two-electrode symmetric capacitor setup was determined from the GCD measurements using the equation,

C (F g^-1^) = $\frac{4*(I\Delta t)}{m\Delta V}$ (2)

E (Wh kg^-1^) = $\frac{C*{\Delta V}^{2}}{2*3.6}$ (3)

P (W kg^-1^) = $\frac{E*3600}{\Delta t}$ (4)

***Battery measurements***

For Li-ion battery electrode preparation, materials were mixed with conductive material acetylene black and mixture of binders polyacrylic acid: carboxymethyl cellulose (1:1) in the ratio 8:1:1 and made into a slurry using ethanol as the solvent. Prepared slurry was coated on Cu foil and dried in a vacuum oven overnight at 100ºC. Li ion battery coin cell was fabricated using CR2032 configuration with active material mass loading in the range of ~ 1.2 - 1.5 mg. Prepared electrode area was 1.539 cm^2^. Thin polypropylene membrane was used as the separator and 400 µL of 1 M LiPF_6_ in ethylene carbonate (EC): diethyl carbonate (DEC): dimethyl carbonate (DMC) (1:1:1 by volume) was used as the electrolyte. After fabricating the coin cell, it was kept for aging for 6 hours and carried out the measurements using Wonatech multichannel battery cycler.

Full cell configuration of the optimized material was prepared with CR2032 coin cell arrangement. ABNC-4 coated on Cu foil as previously mentioned in a half-cell served as the anode. Lithium cobalt oxide (LiCoO_2_) mixed with acetylene black, along with polyacrylic acid and carboxymethyl cellulose mixture in a 95:2.5:2.5 ratio, coated on aluminium foil served as the cathode.

The discharge capacity of the cell is evaluated using the formula:

C_LIB_ (mA h g^-1^) = $\frac{I*t}{m}$ (5)

Where I (mA) is the applied current, t (h) is the discharge time, m (g) is the mass of the active material.

Energy density, E (Wh Kg^-1^) = C_LIB_ * V (6)

Where V (V) denotes the average voltage of the battery.

In the fabricated full cell, ABNC anode mass loading was 1.396 mg and LiCoO_2_ cathode mass was ~ 10 mg. The corresponding N/P ratio is ~1.3. The full cell capacity was calculated by normalizing the measured discharge capacity to the anode’s active material mass.

**Table S1:** Amounts of precursors used for synthesis and the corresponding product yields.

| **Synthesis of nonporous materials** | | | | | | | |
| --- | --- | --- | --- | --- | --- | --- | --- |
| **Material** | **Nonporous CS** | **BNC-1** | **BNC-2** | **BNC-3** | | **BNC-4** | **BNC-5** |
| Boric acid | - | 1 g/16.2 mmol | | | | | |
| Sucrose | 2 g/5.84 mmol | 2 g/5.84 mmol | | | | | |
| Aminoguanidine hydrochloride | - | 1 g/9.05 mmol | 2 g/18.09 mmol | 3 g/27.13 mmol | | 4 g/33.18 mmol | 5 g/45.23 mmol |
| Yield | 0.2 g | 1.68 g | 2.17 g | 2.45 g | | 2.7 g | 3.8 g |
|  | | | | | | | |
| **Solid-state activation** | | | | | | | |
| **Material** | **CS** | **ABNC-1** | **ABNC-2** | **ABNC-3** | **ABNC-4** | | **ABNC-5** |
|  | Nonporous CS: 1 g | BNC-1: 1 g | BNC-2: 1 g | BNC-3: 1 g | BNC-4: 1 g | | BNC-5: 1 g |
|  | PC: 3 g/9.25 mmol | | | | | | |
| Yield | 802 mg | 298 mg | 290 mg | 116 mg | 202 mg | | 175 mg |

**Table S2:** Quantitative estimation of B, C, N, and O using XPS survey spectra and I_d_/I_g_ ratio determined from Raman spectra.

| **Material** | **B** | **N** | **C** | **O** | I_d_/I_g_ |
| --- | --- | --- | --- | --- | --- |
|  |  |  |  |  |  |
| ABNC-3 | 0.03 | 1.9 | 93.26 | 4.87 | 0.95 |
| ABNC-4 | 0.18 | 3.85 | 91.60 | 4.36 | 0.98 |
| ABNC-5 | 0.04 | 5.17 | 89.22 | 5.65 | 0.93 |

**Table S3:** Specific capacitance values of the ABNC-1 and ABNC-2 at different current densities.

|  | **Current density (A g^-1^)** | **0.5** | **1** | **2** | **4** | **5** | **7** | **10** | R_s_  (Ω) | R_ct_  (Ω) |
| --- | --- | --- | --- | --- | --- | --- | --- | --- | --- | --- |
| **Specific capacity (F g^-1^)** | ABNC-1 | 204.76 | 191.20 | 180.46 | 168.51 | 163.76 | 155.76 | 145.00  (71%) | 0.659 | 0.439 |
|  | ABNC-2 | 220.21 | 196.33 | 180.95 | 168.01 | 163.13 | 154.89 | 145.00  (66%) | 0.639 | 0.521 |

**Table S4:** Literature comparison of specific capacitance of similar materials and their textural properties.

| **Material** | **Surface area (m^2^ g^-1^)** | **Pore volume (cm^3^ g^-1^)** | **Electrolyte** | **Specific capacitance (F g^-1^)/ current density (A g^-1^)** | **Capacitance retention%/**  **cycles** | **Energy density (Wh kg^-1^)** | **Power density**  **(W kg^-1^)** | **Ref** |
| --- | --- | --- | --- | --- | --- | --- | --- | --- |
| BNC-850 | 766.5 | 0.6 | 6M KOH | 341.5/0.5 | 95%/10,000 | 126.0 | 459.0 | [1] |
| DHPC-0.5-750 | 2225.3 | 1.17 | 6M KOH | 330/0.5 | **~**99%/20,000 | 40.76 | 450 | [2] |
| NAC (700 ^o^C) | 5770.9 | - | 3M KOH | 210/1.0 | > 80%/1,000 | - | - | [3] |
| BNC-20 | 891 | 0.46 | 6M KOH | 188/0.5 | 91%/10,000 | 9.33 | 400 | [4] |
| BCNC_2.0_-900 | 2991 | 1.39 | 3M KOH | 182.5/1.0 | > 85%/2,000 | - | - | [5] |
| AG10 | 3305 | 1.83 | 3M KOH | 226.5/1.0 | 100%/10,000 | 18 | 582 | [6] |
| HNBC-5K-Zn1 | 3034 | 1.74 | 3M KOH | 247.6/0.5 | 93.8%/5,000 | 33.3 | 666.6 | [7] |
| NGO/CuFe2O4 | - | - | 1M H_2_SO_4_ | 348/1.0 | 87%/2,000 | 35.79 | 883.09 | [8] |
| B-OMC | 301.07 | 0.51 | 6M KOH | 297/0.2 | 88.4%/10,000 | 10.27 | 300.59 | [9] |
| A-MCF | 28.2 | 0.065 | 3M H_2_SO_4_ | 401.4/1.0 | 99.%/20,000 | - | - | [10] |
| **ABNC-4** | **2706.9** | **0.63** | **3M KOH** | **266.5/0.5** | **~ 100%/10,000** | **34.32** | **599.99** | **Current work** |

**Table S5:** Comparison of the stabilized specific capacity of materials at various current densities in LIBs.

| **Current density**  **A g^-1^** | **Capacity (mA h g^-1^)** | | | | | |
| --- | --- | --- | --- | --- | --- | --- |
|  | CS | ABNC-1 | ABNC-2 | ABNC-3 | ABNC-4 | ABNC-5 |
| 0.1 | 178.8 | 521.0 | 910.0 | 1224.1 | 1415.2 | 1115.1 |
| 0.05 | 211.2 | 542.3 | 963.0 | 1299.7 | 1603.1 | 1235.5 |
| 0.1 | 188.3 | 452.7 | 825.7 | 1164.5 | 1440.4 | 1146.8 |
| 0.2 | 164.1 | 363.7 | 695.0 | 994.2 | 1258.4 | 1006.9 |
| 0.5 | 132.1 | 230.2 | 504.2 | 747.5 | 843.3 | 774.6 |
| 1.0 | 93.0 | 135.2 | 289.4 | 513.1 | 459.5 | 520.8 |
| 2.0 | 55.7 | 75.2 | 111.3 | 334.3 | 223.3 | 140.7 |
| 3.0 | 35.9 | 53.5 | 67.6 | 251.9 | 127.4 | 140.7 |
| 0.05 | 213.2 | 519.7 | 948.7 | 1284.8 | 1606.3 | 1234.9 |

**Table S6:** Comparison of resistance values obtained from fitting of Nyquist plots before and after cycling.

|  | **CS** | | **ABNC-3** | | **ABNC-4** | | **ABNC-5** | |
| --- | --- | --- | --- | --- | --- | --- | --- | --- |
|  | **Before** | **After** | **Before** | **After** | **Before** | **After** | **Before** | **After** |
| **R_s_** |  | 2.21 | 3.27 | 2.34 | 2.70 | 3.16 | 2.15 | 4.22 |
| **R_SEI_** |  | 472.6 |  | 92.91 |  | 37.28 |  | 36.78 |
| **R_CT_** | 503.69 | 392.75 | 136.45 | 17.87 | 23.7 | 28.87 | 62.88 | 21.92 |

**Table S7:** Literature comparison of the capacity for some similar reported anodes in lithium-ion batteries.

| **Material** | **Surface area (m^2^ g^-1^)** | **Pore volume (cm^3^ g^-1^)** | | **Capacity (mA h g^-1^)/**  **current density (A g^-1^)** | **Capacitance retention%/**  **cycles** | **Ref** |
| --- | --- | --- | --- | --- | --- | --- |
| B-CN | 480 | | 1.28 | 697/0.1 | - | [11] |
| LGNC 4:1 | 286.8 | | - | 1556/0.1 | - | [12] |
| Si/C-ZIF-8/CNFs | 110.476 | | - | 1334.4/0.1 | 68%/500 | [13] |
| BCN nanoribbons | 995.20 | | 0.23 | 800/0.1 | 98%/1000 | [14] |
| C_60_–BCN-0.05 | 810.5 | | 1.08 | 1164.9/0.1 | ~50%/5000 | [15] |
| WN@BCN-900 | 930.2 | | 1.18 | 683.4/0.1 | 97.3%/500 | [16] |
| N-PCFs | 483.7 | | - | 1437/0.1 | - | [17] |
| N-SPC (Si/C) | 197.9 | | - | 1607/0.4 | 85%/100 | [18] |
| Si@C@void@C |  | |  | 1910/0.1 | 71%/50 | [19] |
| Si/C | 257 | | 0.47 | ~1500/0.1 | ~90%/2500 | [20] |
| SnO-SnO_2_@rGO | 255 | | - | 2604/0.1 | 82%/500 | [21] |
| **ABNC-4** | **2706.9** | | **0.63** | **1396.7/0.1** | **56%/3000** | **Current work** |

**
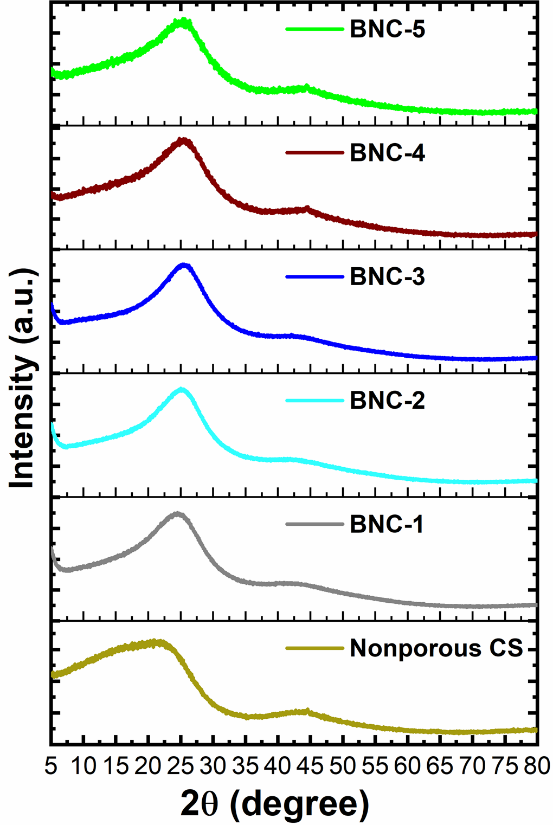
**

**Figure S1.** XRD patterns of non-porous materials.

**
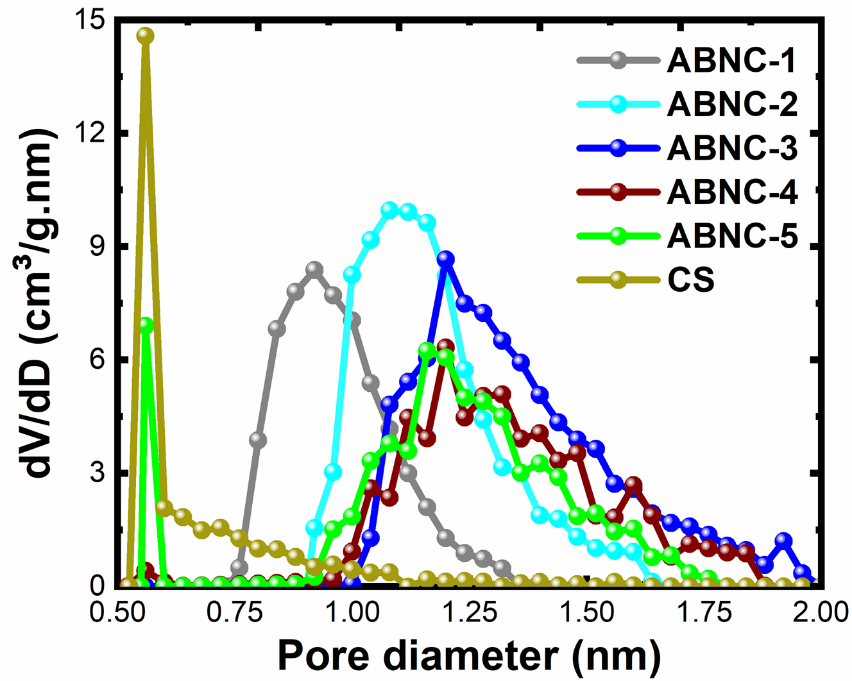
**

**Figure S2.** Pore size distribution by MP method.

**
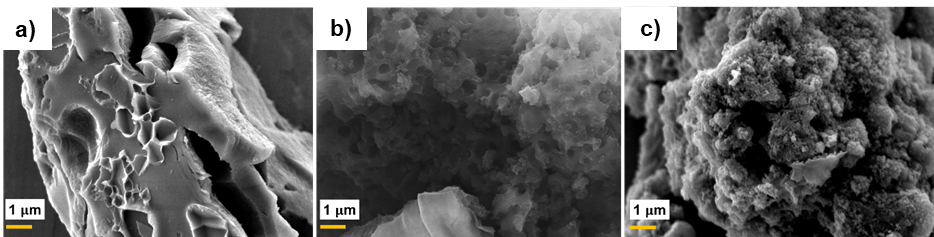
**

**Figure S3.** SEM images of **a)** CS, **b)** ABNC-3, and **c)** ABNC-5.


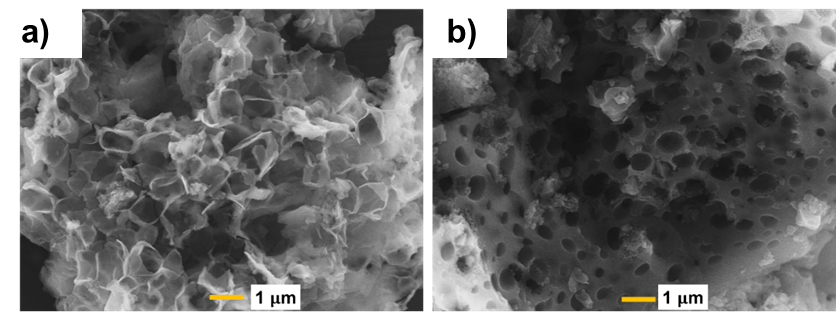


**Figure S4.** SEM images of **a)** ABNC-1 and **b)** ABNC-2.


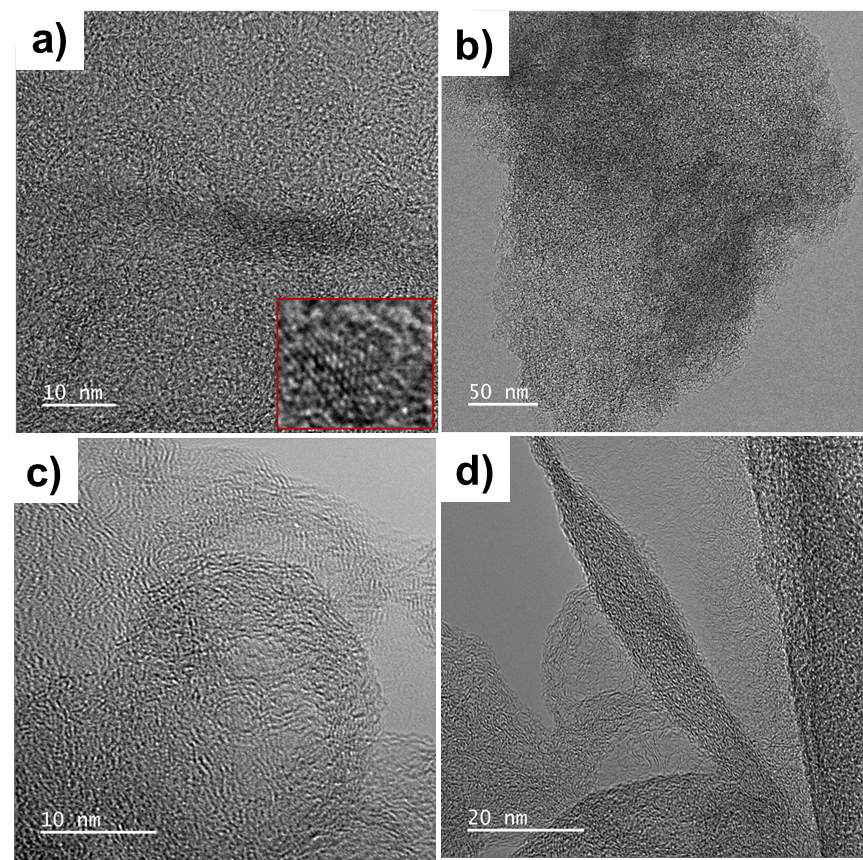


**Figure S5.** TEM images of **a-b)** ABNC-4 and **c-d)** CS at various magnifications.


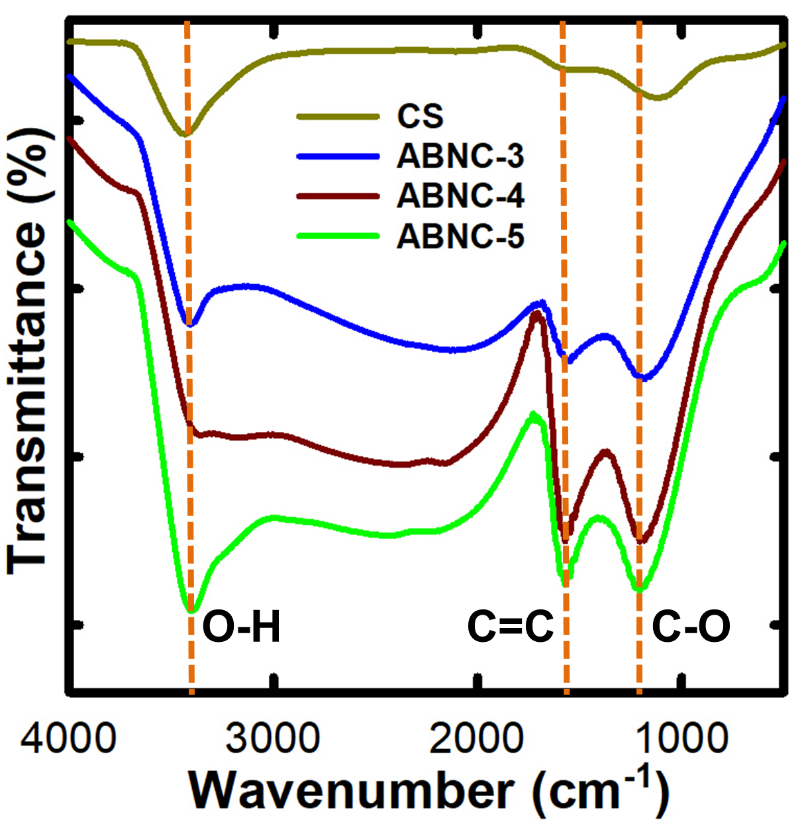


**Figure S6.** FTIR spectra of CS, ABNC-3, ABNC-4, and ABNC-5.


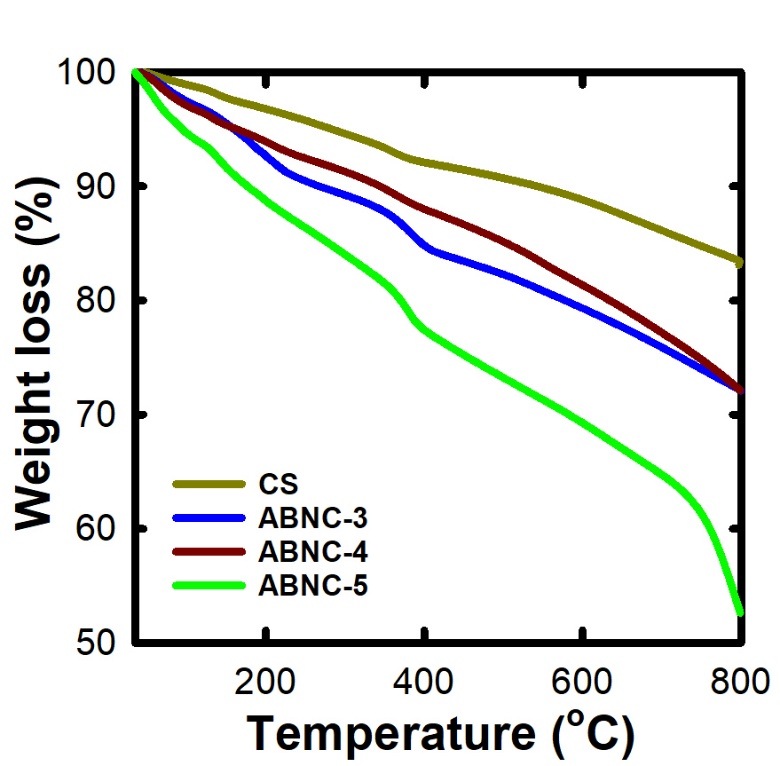


**Figure S7.** TGA analysis of CS, ABNC-3, ABNC-4, and ABNC-5.

**
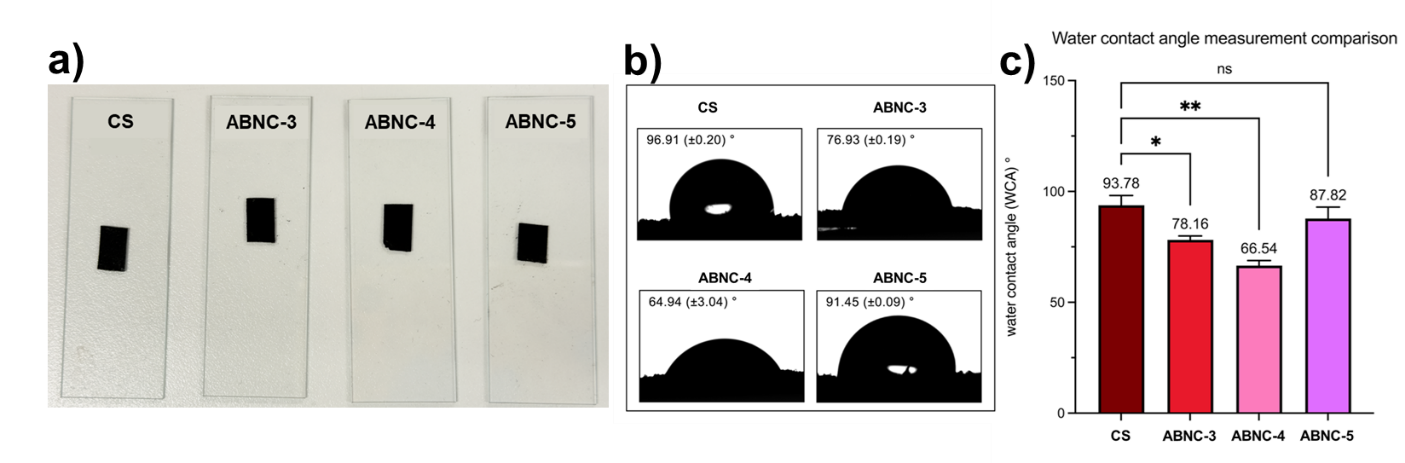
Figure S8. a)** Materials attached on carbon tape for contact angle measurements, **b)** Water contact angle images of materials with Milli-Q water, and **c)** measured angles with standard deviations (*p < 0.05, p <0.01, ns – not significant).


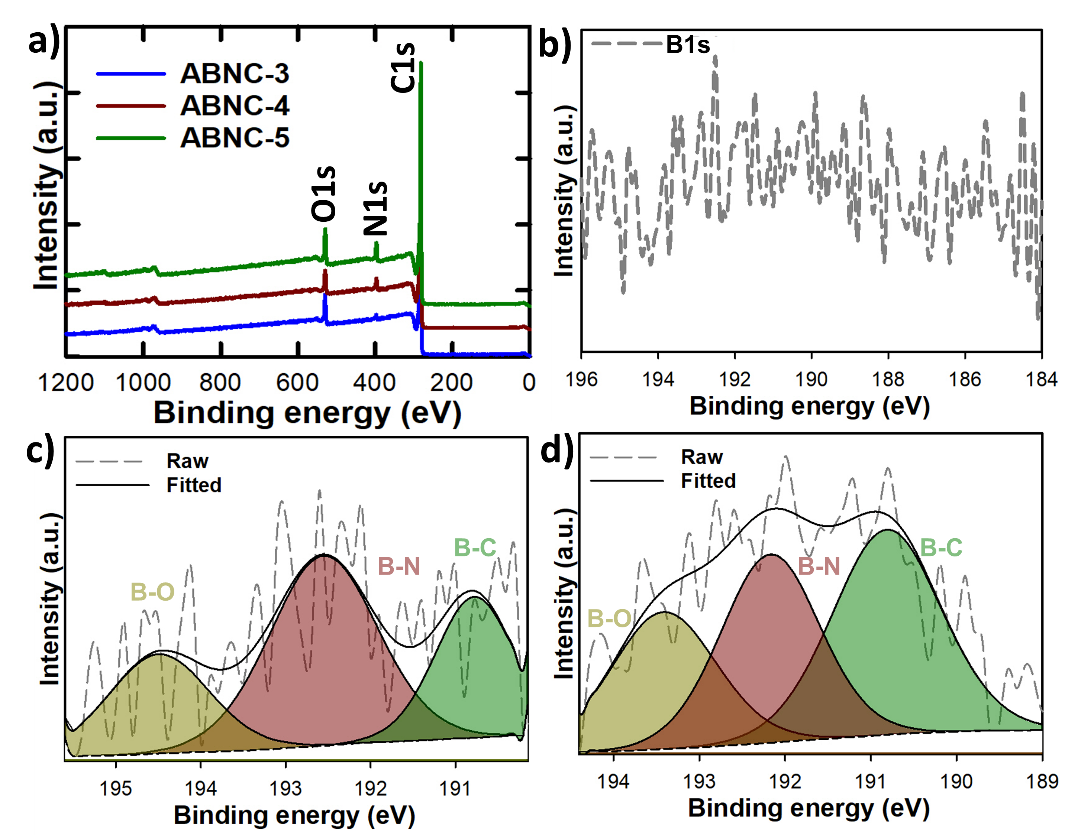


**Figure S9. a)** XPS survey spectra for ABNC-3, ABNC-4, and ABNC-5, High resolution B1s XPS profiles of **b)** ABNC-3, **c)** ABNC-4, **d)** ABNC-5.

**
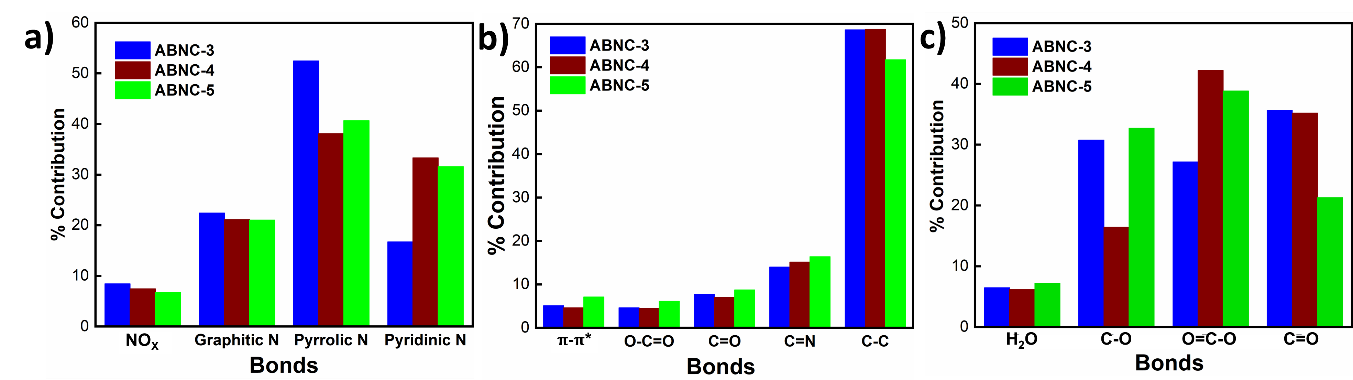
Figure S10.** Contribution of bonds from **a)** N1s, **b)** C1s and **c)** O1s spectra for ABNC-3, ABNC-4, ABNC-5.
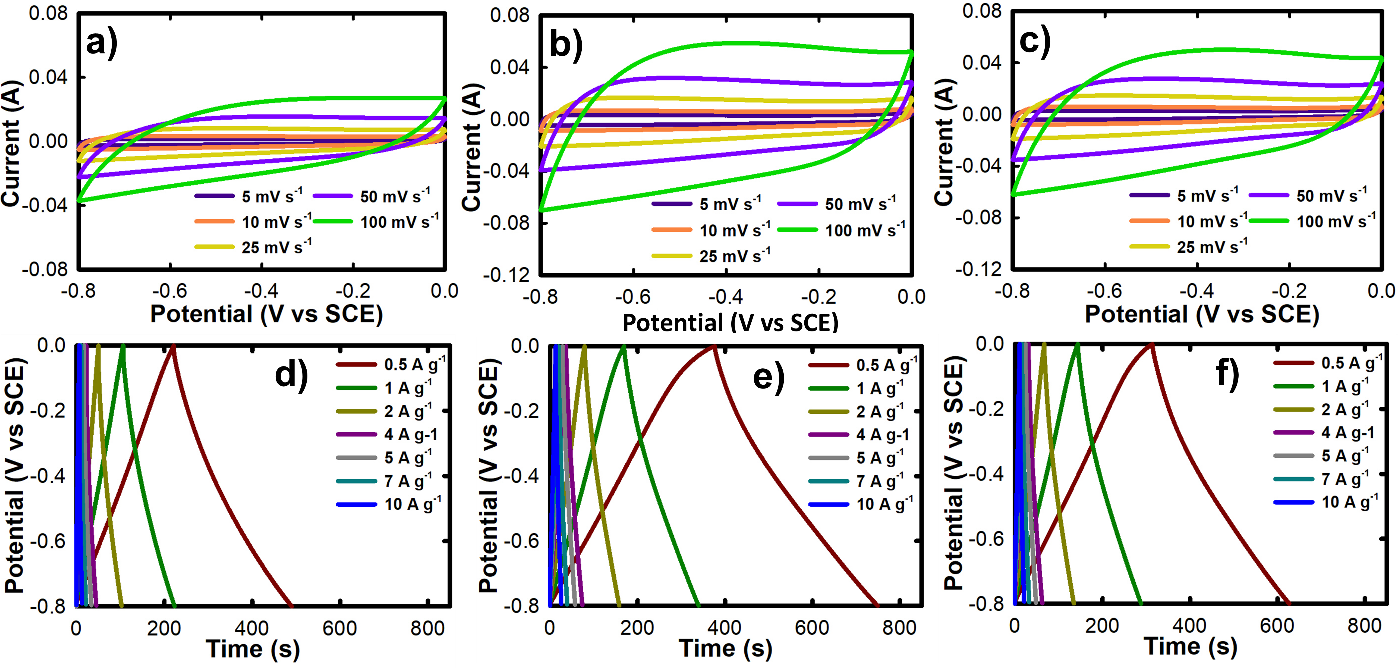
**Figure S11.** CV curves at scan rate 5–100 mV s^-1^ for **a)** CS **b)** ABNC-3 **c)** ABNC-5, GCD curves for the current densities 0.5–10 A g^-1^ for **d)** CS **e)** ABNC-3 **f)** ABNC-5.


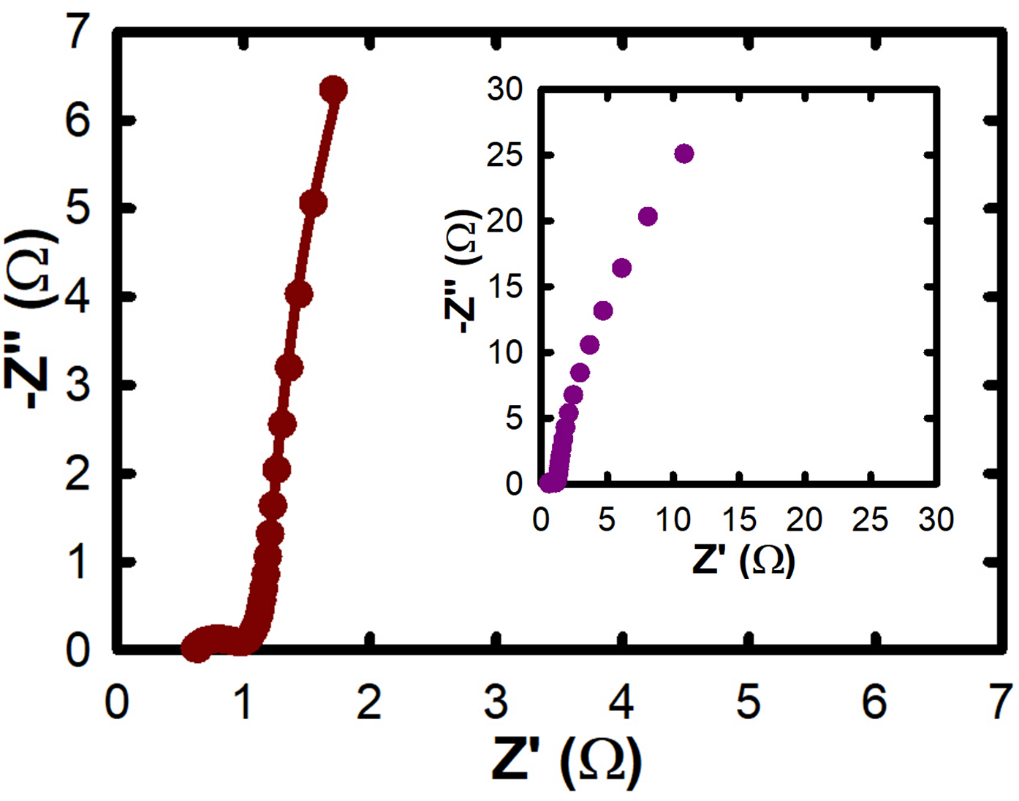


**Figure S12.** Nyquist plot of ABNC-4 at 0.1 - 10^5^ Hz frequency (**inset**: EIS at 0.01Hz – 10^5^ Hz).


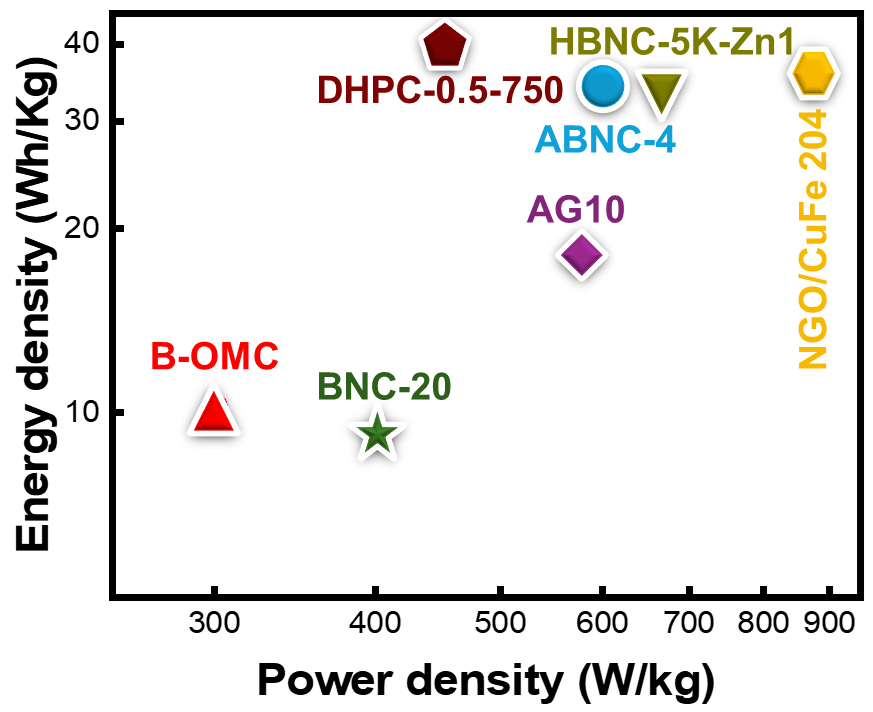


**Figure S13.** Ragone plot comparing the symmetric supercapacitor device with others in literature.


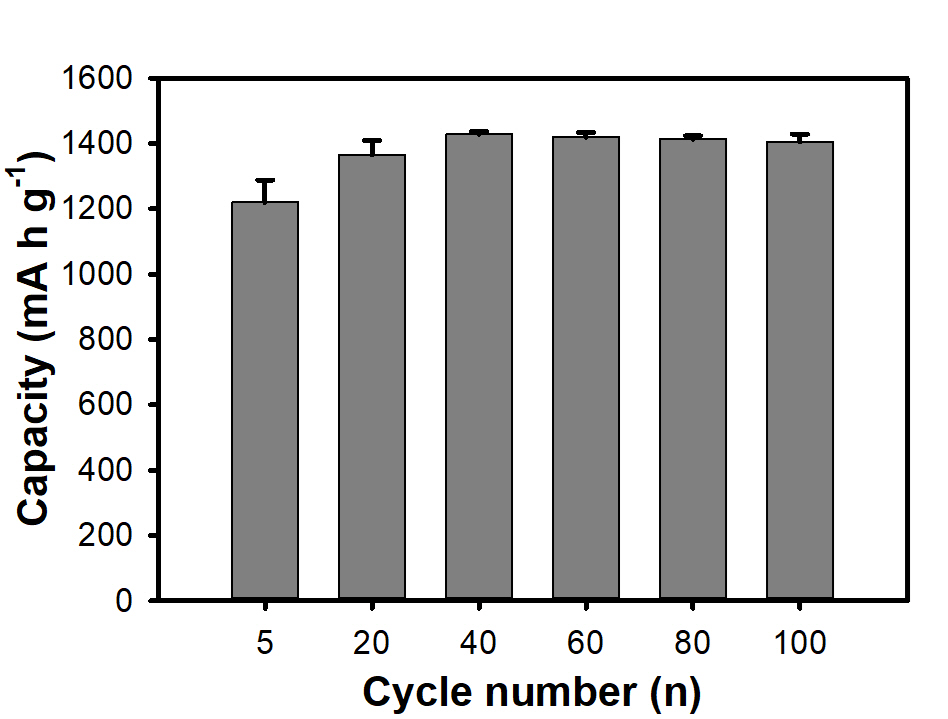


**Figure S14.** Average capacity of ABNC-4 obtained from three different coin cells with corresponding error bars.


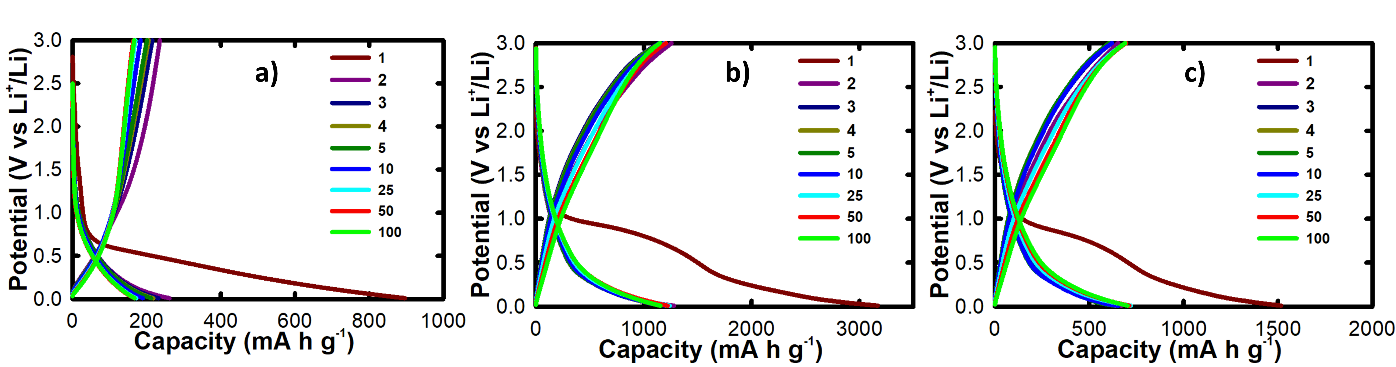


**Figure S15.** Galvanostatic charge-discharge profiles for **a)** CS, **b)** ABNC-3 and **c)** ABNC-5.


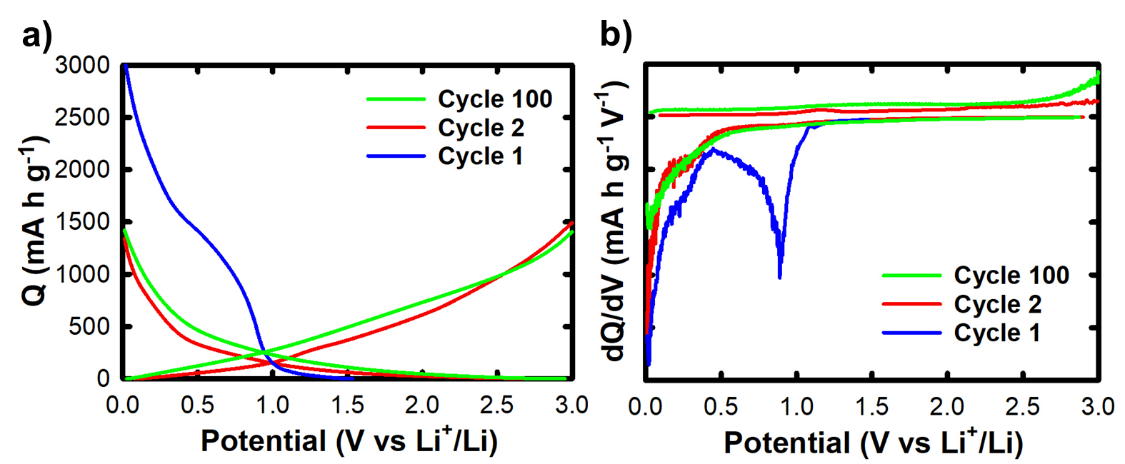


**Figure S16. a)** Galvanostatic charge-discharge profiles and **b)** corresponding differential capacity (dQ/dV) curves of cycle 1, 2, and 100.


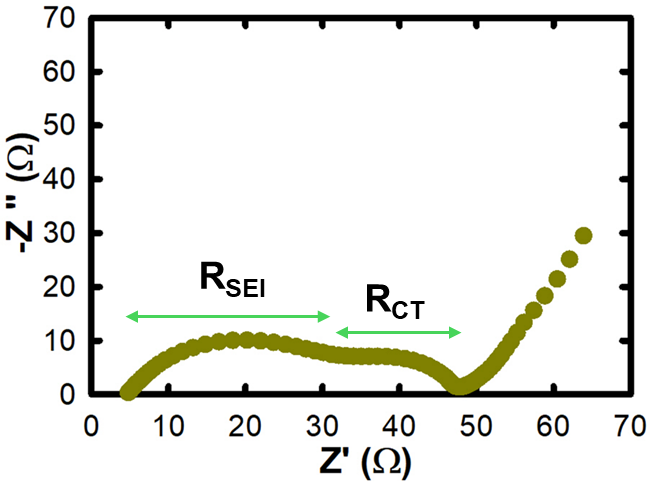


**Figure S17.** Nyquist plot of ABNC-4 LIB coin cell after rate capability measurements.


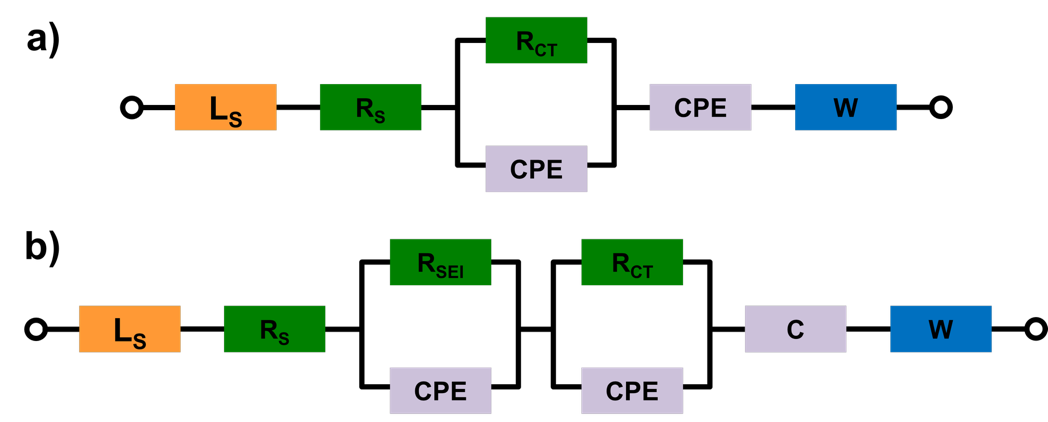


**Figure S18.** Equivalent circuit diagram for Nyquist plots for half-cell **a)** before and **b)** after cycling.


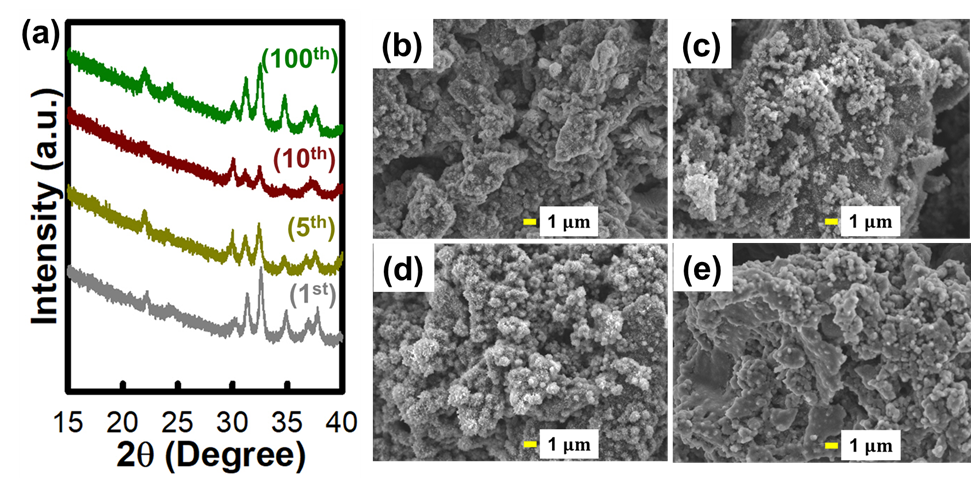


**Figure S19. a)** Ex-situ XRD spectra of ABNC-4 anode after 1^st^, 5^th^, 10^th^, 100^th^ cycles, Ex-situ SEM images after **b)** 1^st^, **c)** 5^th^, **d)** 10^th^, **e)** and 100^th^ cycles.

**
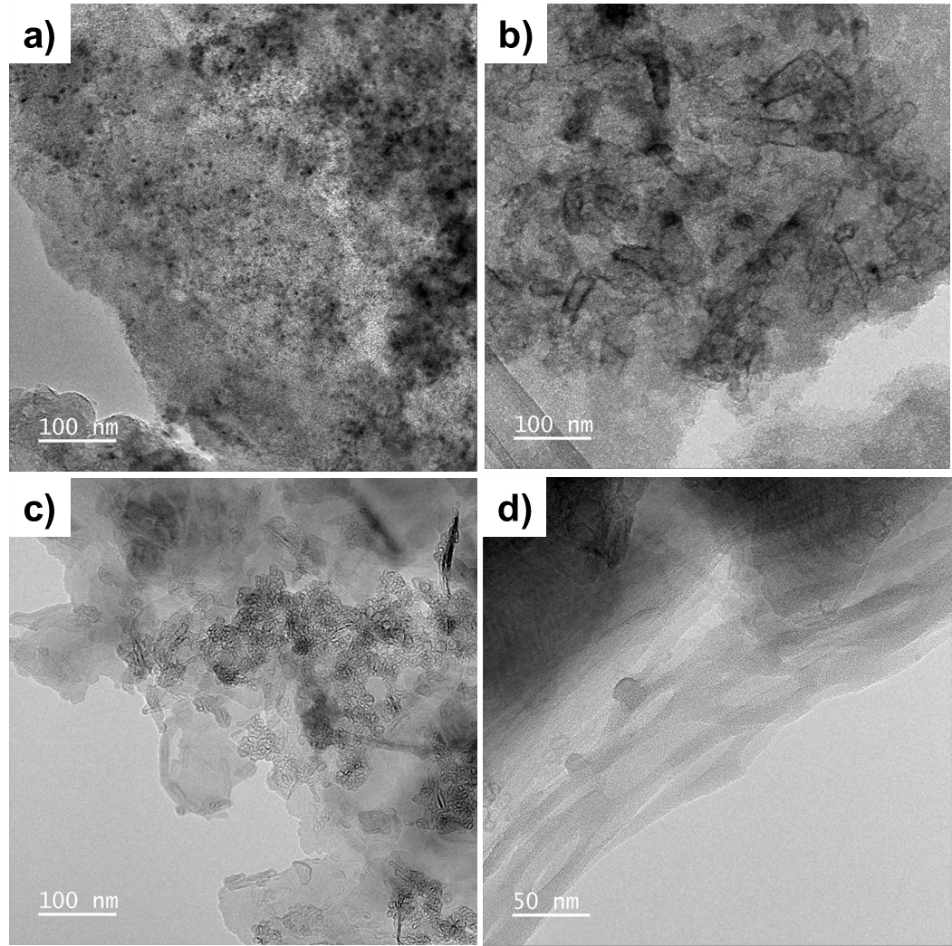
**

**Figure S20.** Ex-situ TEM images of ABNC-4 anode after **a)** 1^st^, **b)** 5^th^, **c)**10^th^, and **d)**100^th^ cycles.


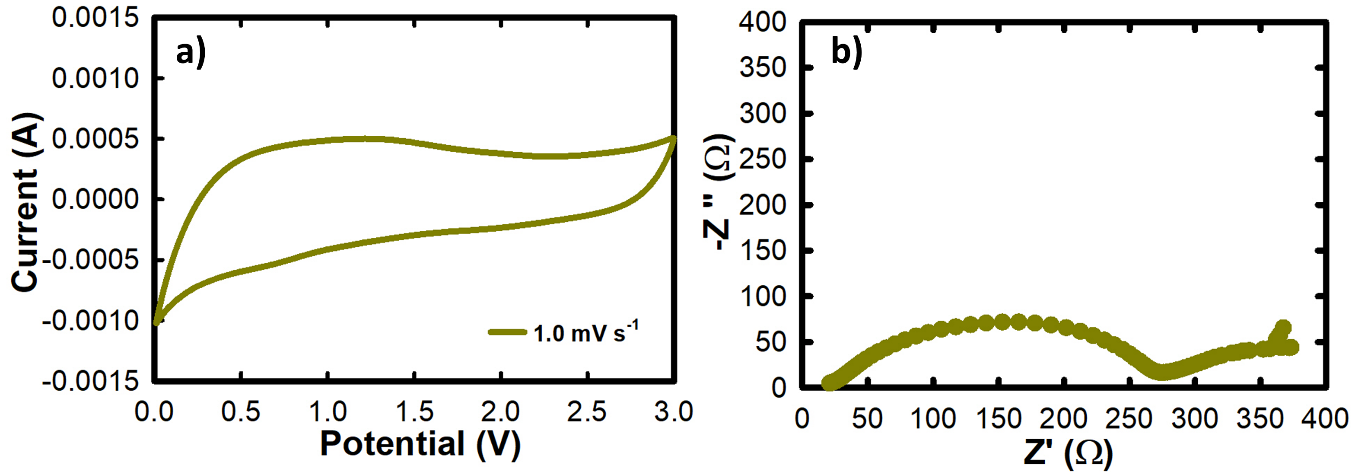


**Figure S21. a)** CV curve at 1 mV s^-1^ and **b)** Nyquist plot of ABNC-4 LIB coin cell after stability measurements.


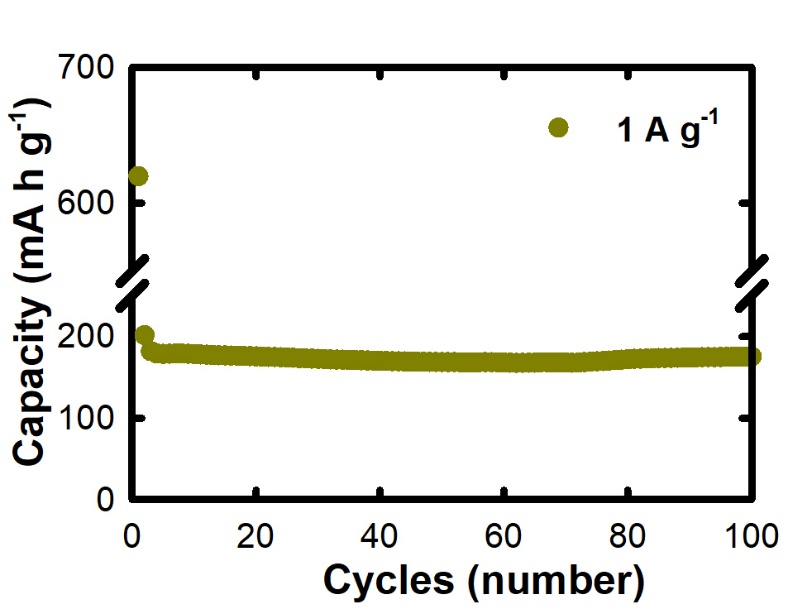


**Figure S22.** Cycling of ABNC-4 coin cell at 1 A g^-1^ in the potential window 0.1 to 3V.

**
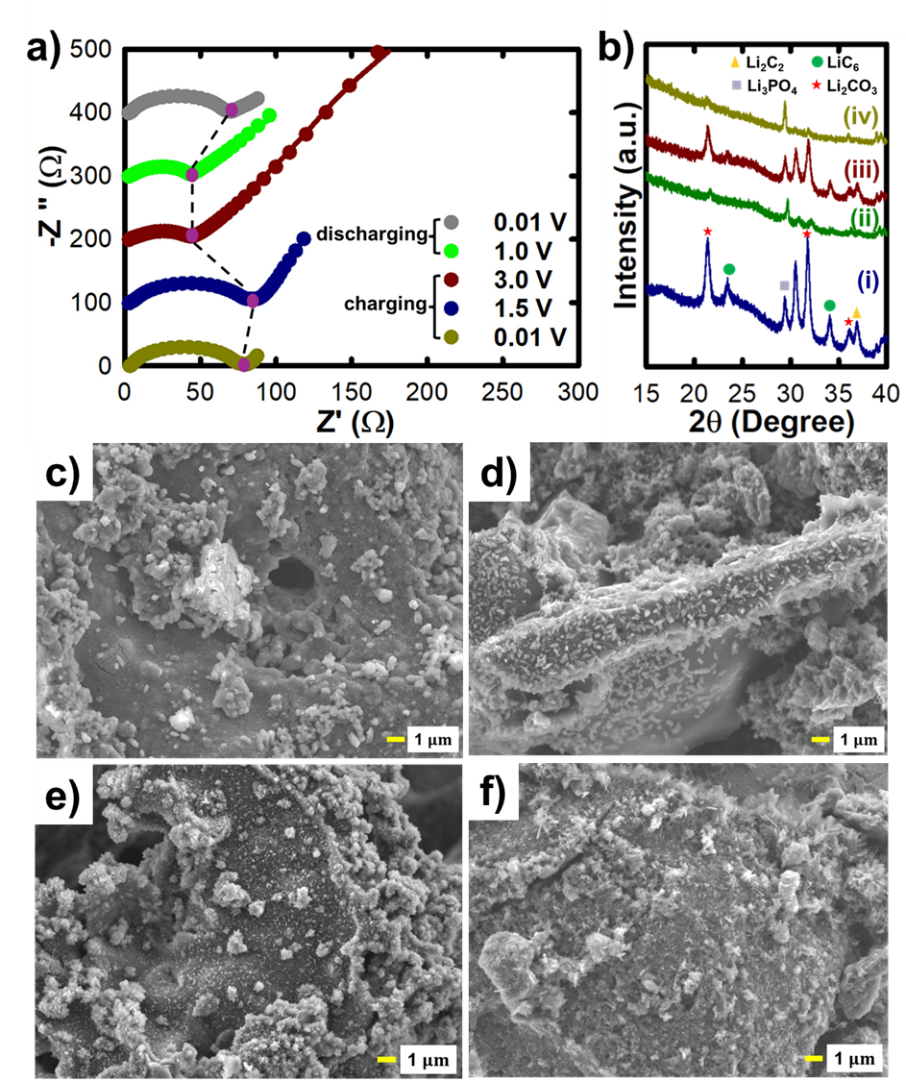
**

**Figure S23. a)** Nyquist plots recorded at different stages of charge-discharge, **b)** XRD spectra at **(i)** 1.5 V, **(ii)** 3 V during charging and **(iii)** 1.0 V, **(iv)** 0.01 V during discharging, SEM images **c)** at 1.5 V during charging, **d)** after complete charging, **e)** at 1.0 V during discharge, and **f)** after complete discharge.

**References**

1. Huang, J.; Peng, J.; Zeng, J.; Zheng, L.; Chen, H., *J. Energy Storage* **2024,** *87*, 111514.

2. Li, H.; Li, Y.; Li, Y.; Shen, H.; Zhu, S.; Zhu, Y.; Lian, K., *J. Energy Storage* **2024,** *77*, 110000.

3. Zimik, M.; Sarmah, S.; Kakati, B. K.; Deka, D.; Thangavel, R., *Mater. Res. Bull.* **2024,** *180*, 113017.

4. Luo, L.; Zhou, Y.; Yan, W.; Wu, X.; Wang, S.; Zhao, W., *Electrochim. Acta* **2020,** *360*, 137010.

5. Bahadur, R.; Singh, G.; Li, M.; Chu, D.; Yi, J.; Karakoti, A.; Vinu, A., *Chem. Eng. J.* **2023,** *460*, 141793.

6. George, N. S.; Bahadur, R.; Fawaz, M.; Tahery, S.; Munroe, P.; Aravind, A.; Sajan, D.; Singh, G.; Vinu, A., *Carbon* **2026,** *246*, 120857.

7. Narayanan, V.; Singh, G.; Ruban, A. M.; Baskar, A.; Bahadur, R.; Perumalsamy, V.; Ramadass, K.; Yang, J.-H.; Mahasivam, S.; Bansal, V.; Vinu, A., *ACS Appl. Mater. Interfaces* **2025,** *17* (27), 39281-39290. DOI 10.1021/acsami.5c09564.

8. Lashkenari, M. S.; Ghasemi, A. K.; Khalid, M.; Shahgaldi, S., *Electrochim. Acta* **2023,** *465*, 142959.

9. Govindarasu, K. G.; Venkatesan, R.; Rajagopal, R. K.; Rajamanickam, G.; Arumugam, P., *Journal of Applied Electrochemistry* **2023,** *53* (6), 1111-1124.

10. Li, K.; Zhang, P.; Soomro, R. A.; Xu, B., *ACS Applied Nano Materials* **2022,** *5* (3), 4180-4186.

11. Wang, D.; Wang, Z.; Li, Y.; Dong, K.; Shao, J.; Luo, S.; Liu, Y.; Qi, X., *Appl. Surf. Sci.* **2019,** *464*, 422-428.

12. Tang, Y.; Chen, J.; Mao, Z.; Roth, C.; Wang, D., *Carbon Energy* **2023,** *5* (2), e257.

13. Zeng, Y.; Huang, Y.; Liu, N.; Wang, X.; Zhang, Y.; Guo, Y.; Wu, H.-H.; Chen, H.; Tang, X.; Zhang, Q., *Journal of Energy Chemistry* **2021,** *54*, 727-735.

14. Chen, S.; Yang, H.; Chen, Q.; Liu, L.; Hou, X.; Luo, L.; Lin, C.; Li, C.; Chen, Y., *Electrochim. Acta* **2020,** *346*, 136239.

15. Bahadur, R.; Singh, G.; Li, Z.; Singh, B.; Srivastava, R.; Sakamoto, Y.; Chang, S.; Murugavel, R.; Vinu, A., *Carbon* **2024,** *216*, 118568.

16. Liu, C.; Wang, C.; Meng, X.; Li, X.; Qing, Q.; Wang, X.; Xue, R.; Yu, Q.; Yang, J.; Wang, K.; Zhao, X.; Chen, W.; Qiao, Z.-A.; Zhao, X. S., *Chem. Eng. J.* **2020,** *399*, 125705.

17. Zhang, X.; Zhang, Z.; Hu, F.; Li, D.; Zhou, D.; Jing, P.; Du, F.; Qu, S., *ACS Sustainable Chemistry & Engineering* **2019,** *7* (11), 9848-9856. DOI 10.1021/acssuschemeng.9b00407.

18. Shao, D.; Tang, D.; Mai, Y.; Zhang, L., *J. Mater. Chem. A.* **2013,** *1* (47), 15068-15075.

19. Xie, J.; Tong, L.; Su, L.; Xu, Y.; Wang, L.; Wang, Y., *J. Power Sources* **2017,** *342*, 529-536. DOI <https://doi.org/10.1016/j.jpowsour.2016.12.094>.

20. Xu, Z.-L.; Gang, Y.; Garakani, M. A.; Abouali, S.; Huang, J.-Q.; Kim, J.-K., *J. Mater. Chem. A.* **2016,** *4* (16), 6098-6106.

21. Kamboj, N.; Debnath, B.; Bhardwaj, S.; Paul, T.; Kumar, N.; Ogale, S.; Roy, K.; Dey, R. S., *ACS nano* **2022,** *16* (9), 15358-15368. DOI 10.1021/acsnano.2c07008.
